# The dynamic interplay of M1/M2-like macrophages and adenosine deaminase activity modulates COVID-19 severity

**DOI:** 10.1515/med-2026-1429

**Published:** 2026-05-04

**Authors:** Tugce Bozkurt, Abdurrahman Simsek, Muhammed Ali Kizmaz, Eren Cagan, Hulya Kose, Ali Eren Iskin, Ayse Melda Payaslioğlu, Mehmet Karadag, Emin Halis Akalin, Sara Sebnem Kilic, Ferah Budak

**Affiliations:** Department of Immunology, Faculty of Medicine, Bursa Uludag University, Bursa, Türkiye; Department of Pediatric Infectious Diseases, Bursa Yuksek Ihtisas Training and Research Hospital, University of Health Sciences, Bursa, Türkiye; Department of Medical Microbiology, Faculty of Medicine, Bursa Uludag University, Bursa, Türkiye; Department of Pulmonary Diseases, Faculty of Medicine, Bursa Uludag University, Bursa, Türkiye; Bursa Uludag University, Faculty of Medicine, Department of Infectious Diseases and Clinical Microbiology, Bursa Uludag University, Bursa, Türkiye; Department of Pediatric Immunology and Rheumatology, Faculty of Medicine, Bursa Uludag University, Bursa, Türkiye

**Keywords:** adenosine deaminase, COVID-19, macrophages, immune dysregulation, flow cytometry

## Abstract

**Objectives:**

The COVID-19 pandemic has highlighted the importance of understanding immune regulation underlying disease severity. Adenosine deaminase (ADA), an enzyme with ADA1 and ADA2 isoforms, modulates immune responses and is mainly expressed in lymphoid tissues. This study examined ADA activity and its relationship with macrophage polarization in COVID-19.

**Methods:**

A total of 120 COVID-19 patients stratified by disease severity and 40 healthy controls were included. Total ADA (tADA), ADA1, and ADA2 activities were measured using the Giusti–Galanti colorimetric method in both patient sera and culture supernatants. Cytokine and chemokine profiles associated with M1- and M2-like macrophages were quantified by ELISA. To mechanistically evaluate macrophage polarization, naïve monocytes were co-cultured with SARS-CoV-2–responsive lymphocytes under a COVID-19 microenvironment (pCOV) generated using viral peptide pools in the presence of COVID-19 patient sera. Macrophage subsets were characterized by flow cytometry using canonical surface markers. The relationship between ADA2 activity and macrophage polarization was functionally assessed using pentostatin, whereas EHNA was used exclusively for the differential measurement of ADA isoenzyme activities.

**Results:**

Elevated total ADA activity was observed in critical patients, correlating with disease severity. Cytokine profiling revealed a hybrid M1/M2-like phenotype in severe cases, characterized by simultaneous pro- and anti-inflammatory mediator release. ADA2 activity showed a strong positive correlation with M2-associated factors, and flow cytometry confirmed the presence of CD206^+^CD86^+^ hybrid macrophages.

**Conclusions:**

Overall, our findings suggest that ADA2 regulates macrophage plasticity in COVID-19, promoting M1/M2 hybrid polarization and contributing to immune dysregulation. ADA2 may serve as a biomarker for disease progression and a therapeutic target to modulate macrophage-driven inflammation in severe COVID-19.

## Introduction

COVID-19, initially identified in Wuhan, China, in December 2019, has emerged as a global pandemic. The causative agent, SARS-CoV-2, can initiate a highly transmissible infection, culminating in severe clinical complications. Although vaccination efforts have reduced disease severity, the virus continues to pose a significant threat by triggering exaggerated immune responses, such as macrophage activation syndrome (MAS), particularly in individuals with pre-existing chronic conditions. MAS constitutes a life-threatening condition, instigating widespread tissue and organ damage. Currently, our comprehension of the impact of macrophage polarization on the progression of COVID-19 severity remains constrained [1], [2], [3], [4].

In the context of COVID-19, particularly in cases marked by a severe clinical presentation, there is a notable decline in lymphocyte count alongside a substantial elevation in C-reactive protein (CRP) and pro-inflammatory cytokines, including IL-6, TNF-α, IL-1β, and IL-8 [5], 6]. The excessive cytokine production observed in COVID-19 cases gives rise to clinical manifestations akin to those seen in macrophage activation syndrome (MAS). Within the cytokine storm, there is a stimulation of various cytokines, including IFN-γ, TNF-α, IL-6, and IL-18, leading to macrophage activation, an intensified immune response, and subsequent multiple organ dysfunction. In response to this scenario, the immune system initiates the secretion of anti-inflammatory cytokines. However, as inflammation persists, there emerge alterations in macrophage responses to diverse stimuli [6], [7], [8].

Adenosine deaminase (ADA; EC 3.5.4.4) is a pivotal enzyme in purine metabolism that catalyzes the irreversible deamination of adenosine to inosine [9], 10]. This enzymatic activity is essential for maintaining immune homeostasis, as adenosine functions as an important immunomodulatory molecule. In humans, ADA activity is mediated by two genetically and structurally distinct isoenzymes: ADA1 and ADA2, the latter also known as cat eye syndrome chromosome region 1 (CECR1) protein [11]. ADA1 is a high-affinity, predominantly intracellular enzyme that tightly regulates intracellular and extracellular adenosine concentrations, thereby preserving purine nucleoside homeostasis. Through this regulatory function, ADA1 prevents the accumulation of lymphotoxic purine metabolites and plays a critical role in modulating T lymphocyte–mediated immune responses. Genetic deficiency of ADA1 leads to the accumulation of toxic metabolites that impair T- and B-cell development, resulting in severe combined immunodeficiency (SCID) [12], [13], [14]. In contrast, ADA2 is a secreted protein with growth factor–like properties primarily derived from cells of the myeloid lineage, including activated monocytes and macrophages. ADA2 has been shown to interact with distinct immune cell populations, including CD16^+^ monocytes as well as B and natural killer (NK) cells, and has been implicated in macrophage polarization and the modulation of adaptive immune responses [15], 16]. Given its central role in immune regulation, alterations in ADA activity – whether through inherited deficiency or acquired dysregulation – have been associated with a broad range of pathological conditions [17], [18], [19]. Beyond SCID, aberrant ADA expression or activity has been implicated in diseases such as tuberculosis [20], 21], systemic lupus erythematosus [22], 23], rheumatoid arthritis [24], coronary artery disease [25], and multiple sclerosis [26], highlighting its relevance in inflammatory and immune-mediated disorders.

Monocyte/macrophage phenotypic alterations can be observed in COVID-19 infection [27]. As the stimuli evolve throughout the inflammatory process, macrophages demonstrate distinct responses. Under normal physiological conditions, a delicate equilibrium is maintained between M1 and M2 macrophages. These macrophage subtypes undergo differentiation in response to changing conditions, aiming to address and rectify the prevailing circumstances [28]. M1 macrophages assume a defensive role by promptly and vigorously responding to pathological conditions. However, their hyperactivation can precipitate chronic inflammatory disorders, including autoimmune diseases. Conversely, M2 macrophages contribute to an anti-inflammatory response, playing a pivotal role in the regulation of the immune system [29].

The infection with SARS-CoV-2 elicits a robust inflammatory response, precipitating the swift release of pro-inflammatory cytokines, notably IL-6, IL-8, TNF-α, and IFN-γ. Investigations have evidenced heightened expression levels of GM-CSF, IFN-γ, IL-6, IL-10, M-CSF, and TNF-α in cases exhibiting mild to moderate severity of COVID-19 in comparison to healthy individuals [30], [31], [32], [33], [34]. Additionally, key signaling molecules such as pulmonary and activation-regulated chemokine (PARC, CCL18) and thymus and activation-regulated chemokine (TARC, CCL17) assume a pivotal role in mediating communication among immune system cells. The levels of these signaling molecules may undergo fluctuations under conditions of inflammation [35], [36], [37]. CCL24, selectively expressed in immune system cells including eosinophils, basophils, and T lymphocytes, serves as a chemoattractant and may exhibit an increase during inflammatory states [38], 39]. Arginase, a critical participant in cellular functions of the immune system, depletes arginine upon activation, thereby influencing cytotoxicity and cellular responses. Additionally, arginase may exert an impact on T lymphocyte proliferation and cytokine production [40], 41]. A comprehensive understanding of the pathogenesis of COVID-19 remains imperative for the development of effective treatment strategies for this complex respiratory ailment.

The aim of this study was to evaluate ADA enzyme activity across different stages of COVID-19 and to examine its relationship with cytokine and chemokine profiles associated with macrophage polarization, thereby providing mechanistic insight into disease severity. We postulated that ADA2 may represent a regulatory factor linking inflammatory signaling and macrophage responses in the progression of COVID-19. Accordingly, we investigated whether ADA isoenzyme activities are associated with disease severity and macrophage polarization–related inflammatory profiles in COVID-19.

## Materials and methods

The study included 120 adult COVID-19 patients (55 women, 65 men; mean age: 54.68 ± 14.93) and 40 healthy controls (24 women, 16 men; mean age: 36.47 ± 10.65). Participants were recruited from Bursa Uludağ University Faculty of Medicine and Bursa Yüksek İhtisas Training and Research Hospital. This study was designed as an observational, severity-stratified clinical investigation to evaluate ADA isoenzyme activity across different stages of COVID-19 in direct relation to disease severity. COVID-19 cases were classified into critical (n=6), severe (n=41), mild/moderate (n=36), and asymptomatic/presymptomatic (n=37) groups based on clinical and laboratory evaluations. All diagnoses were confirmed by RT-PCR testing of nasal and throat swabs. Healthy controls, aged 18–84, were volunteer blood donors with no known diseases or history of COVID-19. Patients were recruited consecutively at the participating centers during the study period according to predefined inclusion and exclusion criteria. All participants provided informed consent, and individuals with comorbidities were excluded. Demographic data are provided in Supplementary Table 1.

### Measurement of ADA enzyme activity

Enzyme activity studies were conducted colorimetrically employing the method delineated by Giusti and Galanti [42]. The assessment of ADA activity in both patient and healthy control groups was executed using a commercially available kit (Elabscience, USA). The protocol for measuring ADA1 and ADA2 activities was uniform. Erythro-9-(2-hydroxy-3-nonyl) adenine (EHNA) (Cayman, USA) was utilized as an inhibitor of ADA1 activity, facilitating the measurement of ADA2-specific enzyme activity.

The calculation of serum ADA1 activity involved the subtraction of ADA2 activity from the total ADA activity. Optimization experiments were conducted for EHNA, determining that a 250 µM EHNA solution was optimal based on existing literature information (Supplementary Figure 1).

### Cell culture

#### Isolation of peripheral blood mononuclear cells (PBMCs)

In the initial phase of our cell culture experiments, PBMCs were isolated from peripheral blood samples obtained from a cohort of healthy controls (n=6). The primary objective of this isolation process was to acquire naïve monocytes and SARS-CoV-2 peptide–responsive lymphocytes for subsequent analyses. PBMCs were isolated from blood samples utilizing the Ficoll (Capricorn, Germany) density gradient centrifugation method.

#### The Percoll density gradient method

The Percoll density gradient method was employed to isolate monocytes from peripheral blood cells and to obtain monocyte-depleted lymphocytes. In the initial phase of the isolation process, hyper-osmotic Percoll (Percoll-Sigma, USA) and iso-osmotic Percoll (Percoll-Sigma, USA) solutions were prepared. To ensure optimal conditions for lymphocyte stimulation, a temporal separation was implemented, with the two stages conducted on different days, specifically 2–3 days prior to monocyte isolation. This temporal arrangement accommodated the 48–72 h required for lymphocyte stimulation.

#### Generation of SARS-CoV-2 peptide–responsive lymphocytes

Following the isolation of PBMCs, cell number and viability were assessed. The lymphocytes were isolated using a hyper-osmotic Percoll solution (Supplementary Figure 2). The isolated lymphocytes were subsequently cultured in RPMI medium supplemented with a pre-optimized mixture of 10 % sera from severe/mild COVID-19 patients (sCOV) at a concentration of 1 × 10^6^ cells per well. Pooled sera (sCOV) were used to minimize inter-individual variability and provide a standardized inflammatory background. The same serum pool was consistently applied within each experimental series to reduce potential batch effects. Samples were treated with a peptide pool containing 88 peptides (9–22 amino acids long) derived from structural (S, M, N, E) and non-structural proteins of the wild type of SARS-CoV-2 (PepTivator, Miltenyi Biotec, Germany). The overlapping peptide design preferentially enriches peptide-responsive CD4^+^ and CD8^+^ T cells rather than inducing broad mitogenic activation. Post-incubation, CD69 expression was analysed in flow cytometry (FC) (Beckman Coulter, France) to assess lymphocyte activation. CD69 was used as an early activation marker to enrich peptide-responsive lymphocytes; however, CD69 positivity was interpreted within the context of peptide and serum conditioning and does not necessarily imply a fully antigen-experienced phenotype.Sample groups with less than 50 % activation and those with less than 95 % cell viability were excluded from further analysis (Supplementary Figure 3). This activation threshold was applied to ensure consistent enrichment of peptide-responsive populations while maintaining high cellular viability across experimental replicates. Control lymphocytes, essential for subsequent stages, were stimulated with LPS [5 μg/mL, (Thermo Fischer, USA)] for M1 control and IL-4 [20 ng/mL, (Thermo Fischer, USA)] for M2 control, both in the presence of 10 % autoserum and PHA. Unstimulated (US) control lymphocytes received no additional agents. All control lymphocytes and SARS-CoV-2 peptide–responsive T cells were cultured under equivalent conditions for 2–3 days.

#### Isolation of monocytes

The isolation of the monocytes was initiated 2–3 days after the start of the T-cell culture. The second isolation of PBMCs was conducted by obtaining fresh blood samples (for the second time). Monocytes were harvested from PBMCs using both hyper-osmotic Percoll and iso-osmotic Percoll solutions. Cell counting was performed and cell viability was confirmed, excluding samples with less than 95 % viability from further analysis. After cell counting, the purity of CD14-expressing cells was assessed by FC (Supplementary Figure 4). Samples with a purity of less than 85 % were not subjected to further analysis.

#### Co-culturing SARS-CoV-2-specific lymphocytes with naive monocytes

SARS-CoV-2 peptide–responsive lymphocytes, control lymphocytes (PHA-induced), unstimulated lymphocytes, and naïve monocytes were co-cultured in a 1:2 ratio of lymphocytes to monocytes. The stimulation of SARS-CoV-2 peptide–responsive lymphocytes and monocytes was carried out using sCOV, which consists of a mixture of peptide pools. To mitigate potential confusion, it is essential to emphasize that, within the context of this study, the *in vitro* microenvironment for COVID-19, formed using the peptide pool and sCOV, is consistently identified as p-COV throughout the manuscript. Three comparison groups were established: M1-control (LPS+GM-CSF-stimulated), M2-control (IL-4+M-CSF-stimulated), and US groups. PHA was added to all groups except US group to stimulate T cell proliferation. The co-cultures were incubated at 37 °C and 5 % CO_2_ for 9–11 days. The medium was refreshed every 3–4 days, and the stimulations were repeated. The morphological characteristics of the macrophages were monitored regularly using phase-contrast microscopy. After the co-culture period, the cells were harvested for FC analysis, and the supernatants were stored at −80 °C for ELISA analysis.

#### Inhibition of ADA with pentostatin and EHNA

To evaluate the direct effect of ADA1 and ADA2 enzyme activity on macrophage polarization, the chemotherapeutic agent pentostatin (2′-deoxycoformycin) (Sigma-Aldrich-USA) was used. Due to its known potent cytotoxic effects, various concentrations ranging from 0.5 μmol/L to 50 μmol/L were tested under the specified co-culture conditions. The optimal concentration, which ensured the survival of T cells and monocytes while achieving optimal ADA inhibition (with an activity ratio of less than 20 %), was found to be 10 μmol/L. EHNA (250 μM) was similarly applied, though it exclusively targets ADA1, in contrast to pentostatin’s dual inhibition. Consequently, EHNA-induced total ADA (tADA) activity showed only a minimal reduction (70–80 % tADA activity ratio) compared to the control group. The previously detailed co-culture model was reconstituted in parallel for both inhibitor arms.

### Assessment of M1 and M2-like macrophage polarization by FC

The polarization of macrophages was assessed using FC following the co-culture of SARS-CoV-2 peptide–responsive lymphocytes and naïve monocytes. For each co-culture, three flow cytometer tubes were prepared, one of which served as an isotypic control. A volume of 100 µL of cells was added to each tube, with isotypic control antibodies in the first tube, propidium iodide as a viability marker in the second tube, and CD45 (Kro, Beckman Coulter, France), CD14 (A700, Beckman Coulter, France), CD80 (APC, Beckman Coulter, France), CD86 (A750, Beckman Coulter, France), CD200R (PE, Biolegend, USA) and CD206 (PB, Beckman Coulter, France) in the third tube for the analysis of macrophage subgroups.

**Figure 1:**
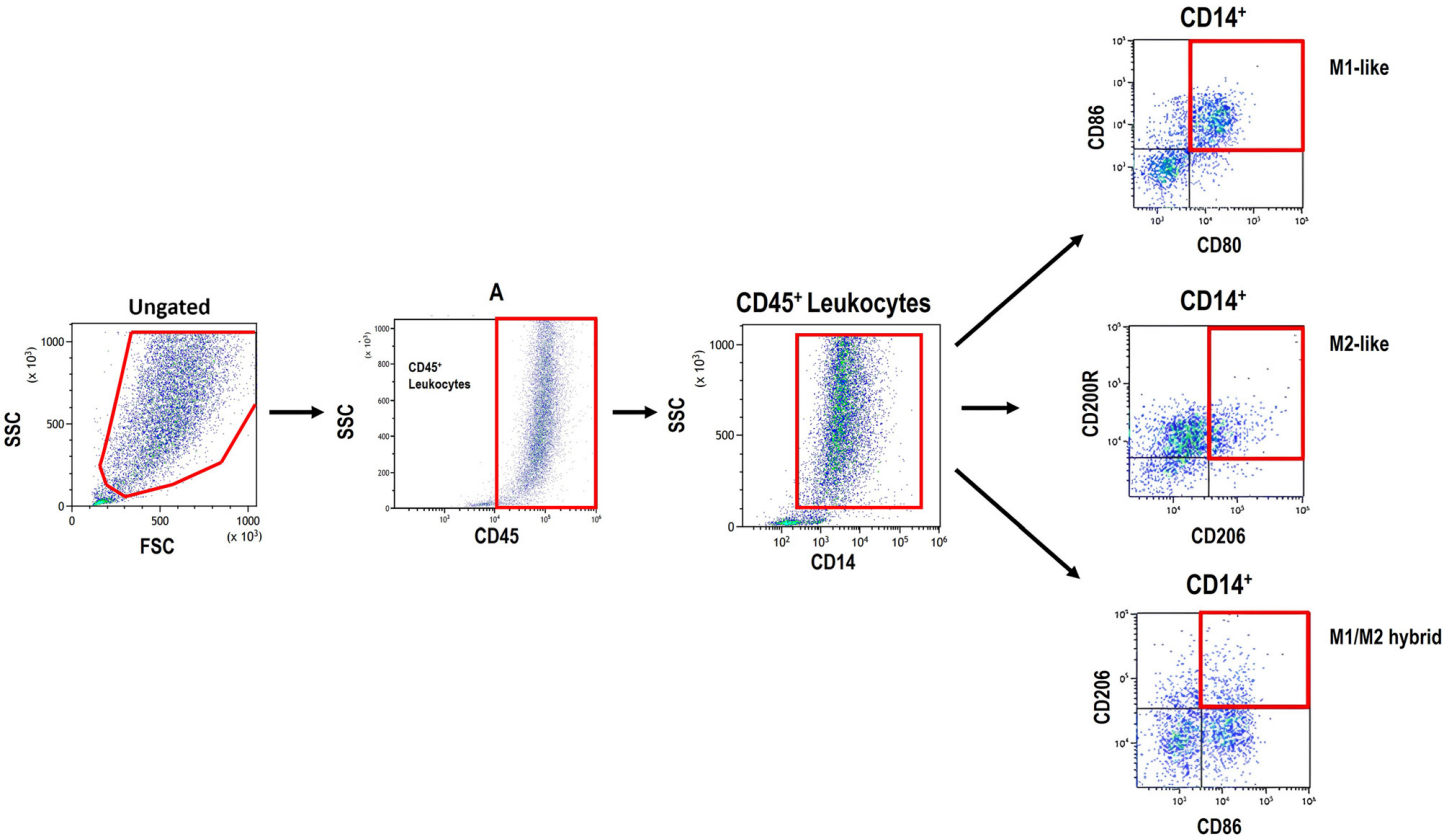
Flow cytometry gating strategy for investigating macrophage polarization.

The basic gating strategy to identify macrophage subsets is illustrated in Figure 1. Debris was excluded using the SS and FS parameters. CD45^+^ leukocytes and CD14^+^ monocytes/macrophages were gated accordingly. Macrophages were further classified into M1-like macrophages (CD80^+^CD86^+^), M2-like macrophages (CD200R^+^CD206^+^), and hybrid M1/M2 macrophages (CD86^+^CD206^+^) based on their expression of CD80, CD86, CD200R, and CD206.

### Determination of M1 and M2 related factors by ELISA

A total of 48 patients were enrolled in this phase of the study, including 6 from the critically ill group, 14 classified as severe, 14 as mild/moderate, and 14 as presymptomatic. Additionally, 14 participants from the healthy control group were included. Post-co-culture supernatants from pCOV and control groups were also incorporated. M1-related factors (IL-6, IL-12, TNF-α, IL-18, IFN-γ, GM-CSF, CXCL-10, ROS, iNOS) and M2-related factors (IL-4, IL-10, M-CSF, PARC (CCL18), TARC (CCL17), CCL24, CXCL-9, Arginase) were measured using commercial ELISA kits (Sunred Bio-Technology, China). The experiments were conducted following the recommended protocol provided by the commercial kit manufacturer.

### Statistical analysis

In this study, statistical analyses were performed using IBM SPSS Statistics (Version 28.0), Python (v3.10), GraphPad Prism (Version 9.5.1), and Origin Pro (Version 2023b). Python was used for multivariable logistic regression modeling and stratified 5-fold cross-validation, utilizing the *pandas*, *statsmodels*, and *scikit-learn* libraries. Receiver operating characteristic (ROC) curves and area under the curve (AUC) values were computed in Python, while graphical representations were generated using *matplotlib*, GraphPad Prism (v.10), and Origin Pro (2023b). Distribution normality was assessed visually (histograms, probability charts) and analytically (Kolmogorov–Smirnov, Shapiro–Wilk tests). For parametric data, multiple group comparisons were performed using ANOVA with post-hoc Tukey HSD test; for non-parametric data, Kruskal–Wallis test with post-hoc Dunn test was used for multiple comparisons and Mann-Whitney test for pairwise comparisons. Categorical variables were compared using χ^2^ or Fisher’s exact test. Pearson correlation test was used for parametric data, with correlation coefficients (R) interpreted as weak (0–0.3), moderate (0.3–0.7), or strong (0.7–1.0), and statistical significance set at p<0.01. An *a priori* power analysis was performed using G*Power (Version 3.1) with 80 % power and two-sided α=0.05, targeting medium-to-large within-subject effects, and effect sizes (eta-squared, partial eta-squared) were calculated using standardized measures. To address potential confounders, multivariable logistic regression was performed to assess the independent association between ADA2 activity and mortality, including clinically relevant covariates (age, sex, CRP, ferritin, D-dimer) selected *a priori*. Odds ratios with 95 % confidence intervals were calculated, and model discrimination was evaluated using ROC curve analysis. To reduce optimism bias, stratified 5-fold cross-validation was applied, with cross-validated AUC values reported as mean ± SD Statistical significance was defined as p<0.01 for all analyses.

### Ethical approval

The study protocol was reviewed and approved by the Clinical Research Ethics Committee of Bursa Uludağ University Faculty of Medicine (Decision No: 2020-16/10; Date: 16 September 2020). The study was conducted in accordance with the principles of the Declaration of Helsinki. All participants were informed about the purpose and procedures of the study, and written informed consent was obtained prior to enrollment. Participation was entirely voluntary, and participants were free to withdraw at any stage without consequence. All collected data were anonymized and handled confidentially in compliance with institutional and national data protection regulations.

### Consent to participate

Informed consent forms were obtained from all participants.

## Results

### Enhanced activity of ADA1 and ADA2 enzymes in severe and critical COVID-19 cases

**Figure 2:**
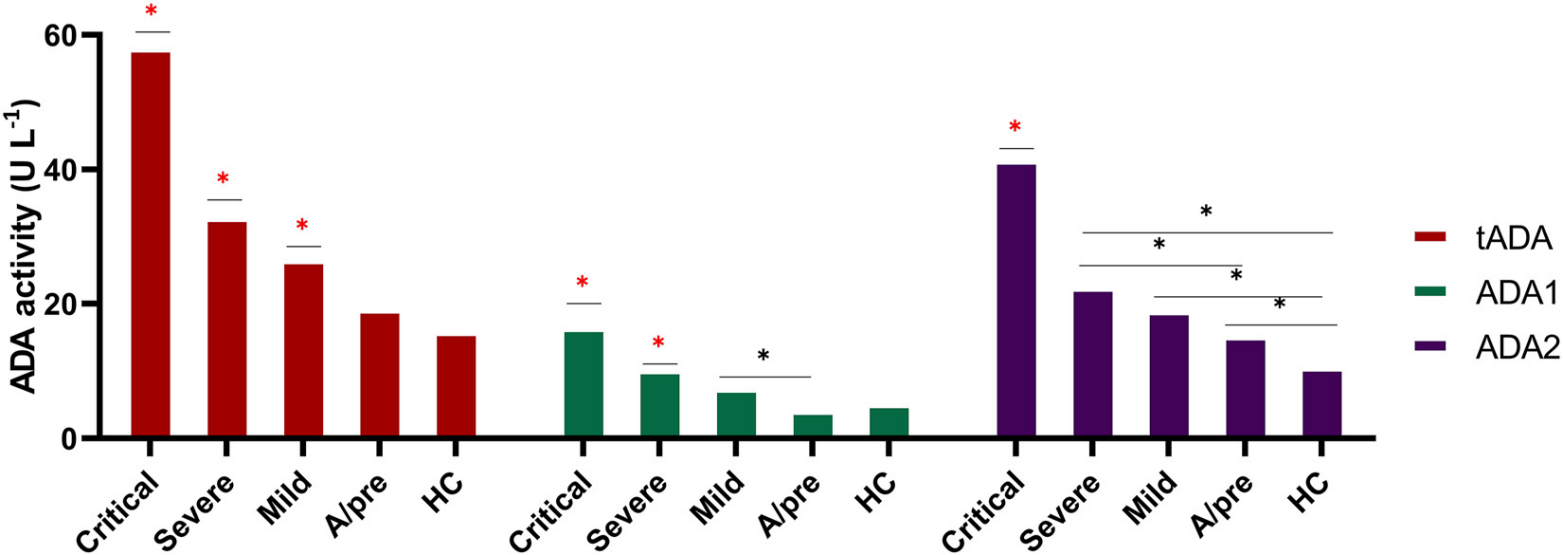
ADA activity elevates in COVID-19 patient groups: ‘*’ denotes statistical significance at p<0.005. The ‘
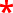
’ highlighted in red defines a statistically significant difference from all groups. For multiple comparisons, ANOVA followed by post hoc Tukey’s HSD test was applied. Data are presented as mean ± SEM. Sample sizes are: critical (n=6), severe (n=41), mild (n=36), A/pre (n=37), and HC (n=40).

The assessment of tADA, ADA1, and ADA2 activities demonstrated a substantial elevation in critical and severe cases when compared to other case groups (Figure 2). When expressed as a percentage, ADA2 activity emerges as predominant, constituting 72 % in critical COVID-19 cases and 68 % in severe COVID-19 cases (Supplementary Figure 5). The upregulation of ADA1 was comparatively modest, whereas ADA2 exhibited a statistically significant increase even in presymptomatic individuals.

### ADA2 activity exhibits heightened response to pCOV

**Figure 3:**
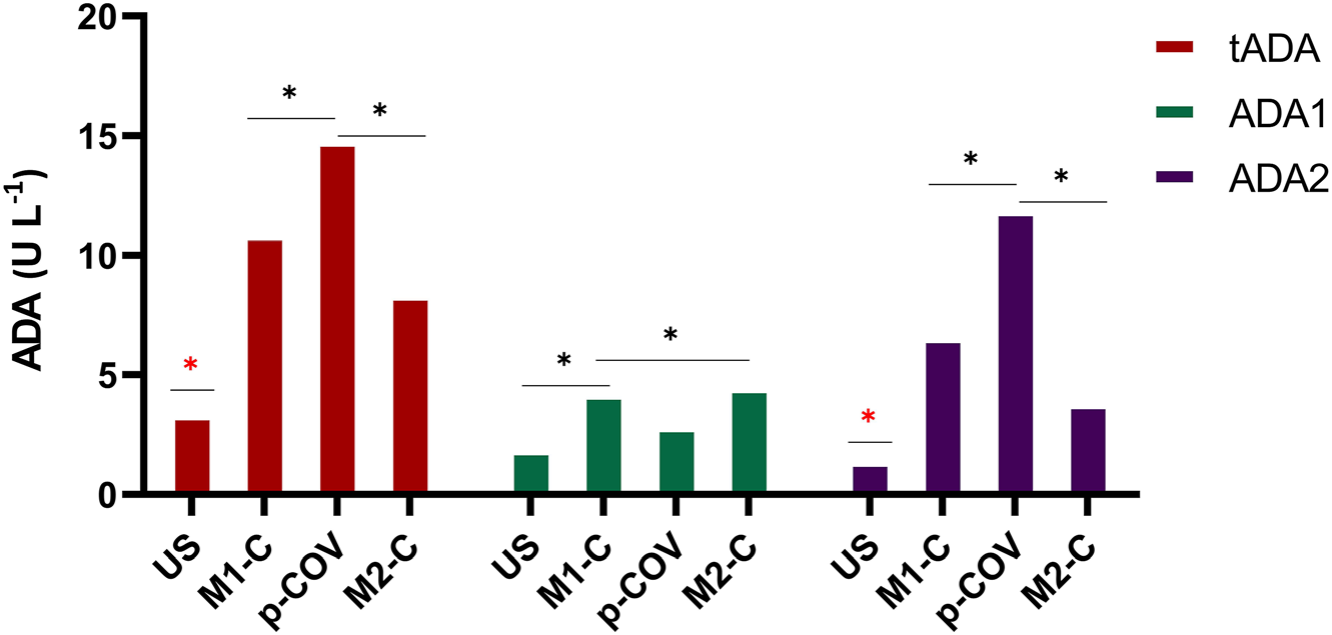
After co-culture experiments, the highest level of ADA2 activity was observed in the p-COV group. ‘*’ denotes statistical significance at p<0.005. The ‘
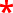
’ highlighted in red defines a statistically significant difference from all groups. For multiple comparisons, ANOVA followed by post hoc Tukey’s HSD test was applied. Data are mean ± SEM. Results represent independent experiments using cells from different healthy donors (n=6) for each condition (US, M1C, M2C, and pCOV).

Analysis of ADA activity in cell culture experiment supernatants revealed a significant overall increase, most prominently in ADA2 activity, which reached 79.1 % in the p-COV group (Figure 3 and Supplementary Figure 6). Consistent with serum ADA2 activity patterns, cell culture experiments demonstrated the highest ADA2 levels in the p-COV group, whereas ADA1 activity was comparatively reduced relative to both M1 and M2 control groups.

### M1 and M2 cytokine and chemokine profiles mirror disease severity progression

**Figure 4:**
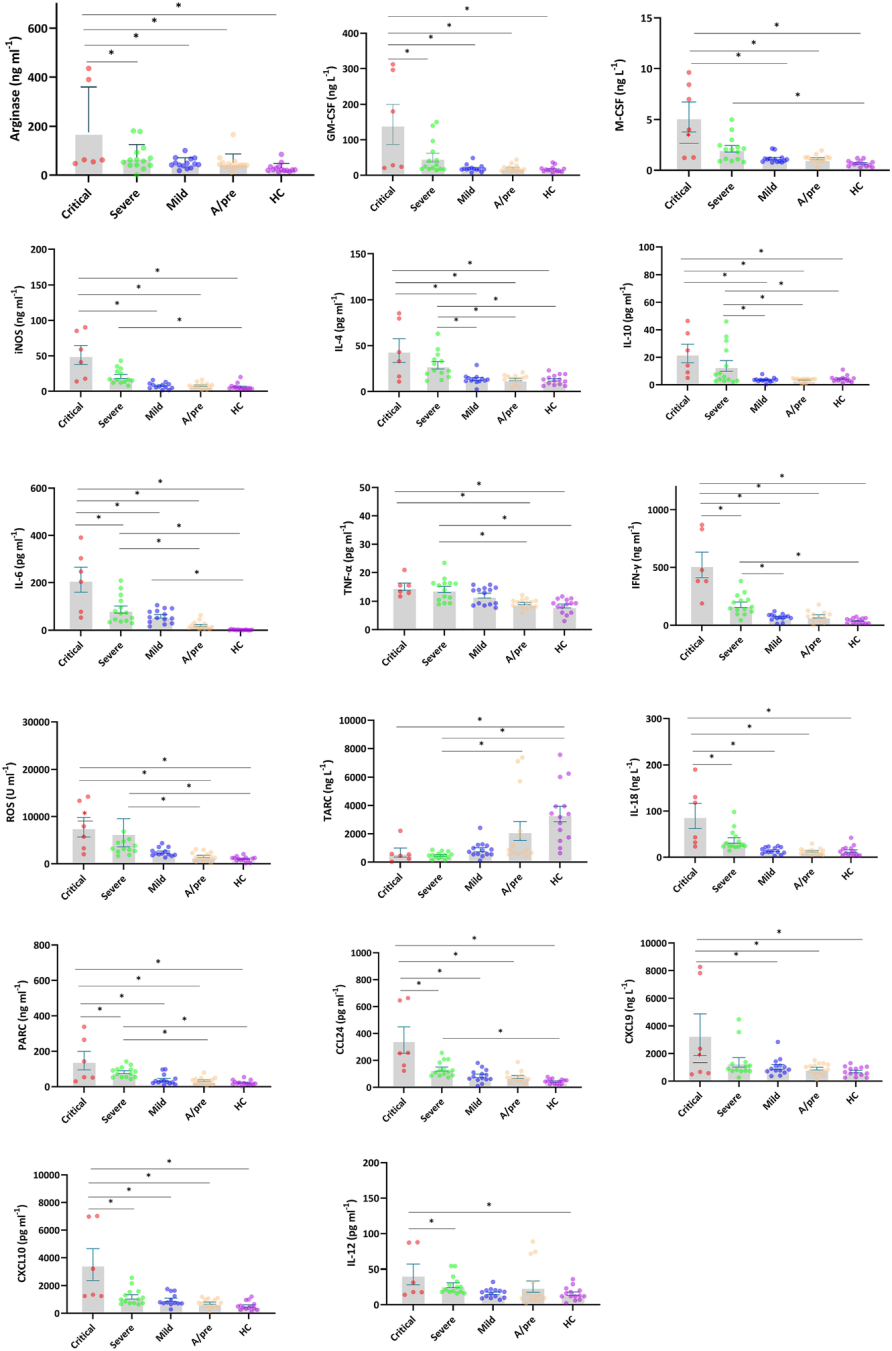
Levels of M1 and M2-related factors in COVID-19 patient groups. ‘*’ denotes statistical significance at p<0.005. For multiple comparisons, ANOVA followed by post hoc Tukey’s HSD test was applied. Data are presented as mean ± SEM. Sample sizes are: critical (n=6), severe (n=41), mild (n=36), A/pre (n=37), and HC (n=40).

In our study, notable increases in pro-inflammatory markers, including IL-6, TNF-α, IL-12, IFN-ɣ, GM-CSF, ROS, iNOS, cytokines, and factors associated with M1 activation, were observed in critical and severe cases. Intriguingly, higher levels of Arginase, IL-4, IL-10, and M-CSF, which are indicative of M2 activation, were also noted in critical and severe cases (Figure 4).

In addition, among the chemokines, CXCL9 and CXCL10 exhibited a clear upward trend in the critical group compared with the severe group, reflecting a directional increase as disease severity escalated, although not all pairwise comparisons achieved statistical significance. Similarly, IL-18 levels followed an increasing tendency in correlation with clinical severity. In contrast, TARC levels showed a downward trend in the critical group relative to other categories, representing a decreasing pattern as the disease progressed.

### Signature profile of cytokines and chemokines in the pCOV

**Figure 5:**
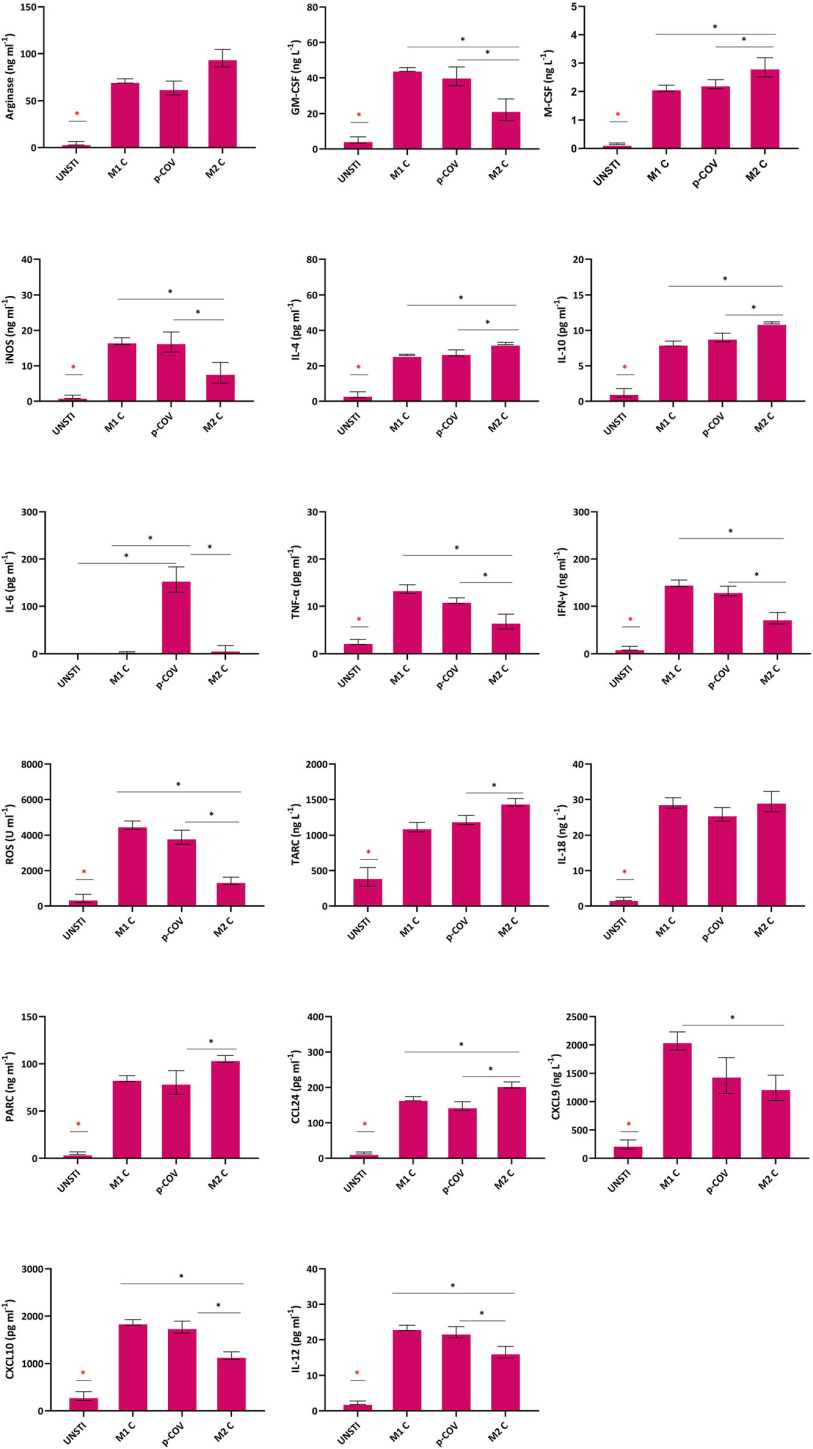
Levels of M1 and M2-related factors were assessed in supernatants obtained from coculture experiments. ‘*’ denotes statistical significance at p<0.005. For multiple comparisons, ANOVA followed by post hoc Tukey’s HSD test was applied. Data are mean ± SEM. Results represent independent experiments using cells from different healthy donors (n=6) for each condition (US, M1C, M2C, and pCOV).

In our cell culture experiments, a comparative analysis of the pCOV group revealed that M1-related proinflammatory cytokines, chemokines, and other factors exhibited a profile similar to the M1-control group. Upon analysis of the pCOV, M1-related factors, encompassing IL-6, IL-12, TNF-α, IFN-ɣ, GM-CSF, CXCL10, ROS, and iNOS, displayed elevated levels compared to the M2 control group, closely mirroring the profile observed in the M1 control group. Conversely, M2-related cytokines and chemokines, specifically IL-4, IL-10, CCL18 (PARC), CCL17 (TARC), CCL24, and arginase, exhibited a reduction in the pCOV compared to the M2 control group (Figure 5). Additionally, IL-6 was found to be significantly higher in the COVID-19 group compared to other groups. From a holistic standpoint, the pCOV group exhibited intermediary levels between M1 and M2 controls in both pro-inflammatory and anti-inflammatory cytokines, chemokines, and other factors. This pivotal observation provides compelling evidence suggesting that both M1 and M2 activations may contribute to the severity of COVID-19.

### Heterogeneous macrophage populations in severe and critical COVID-19

**Figure 6:**
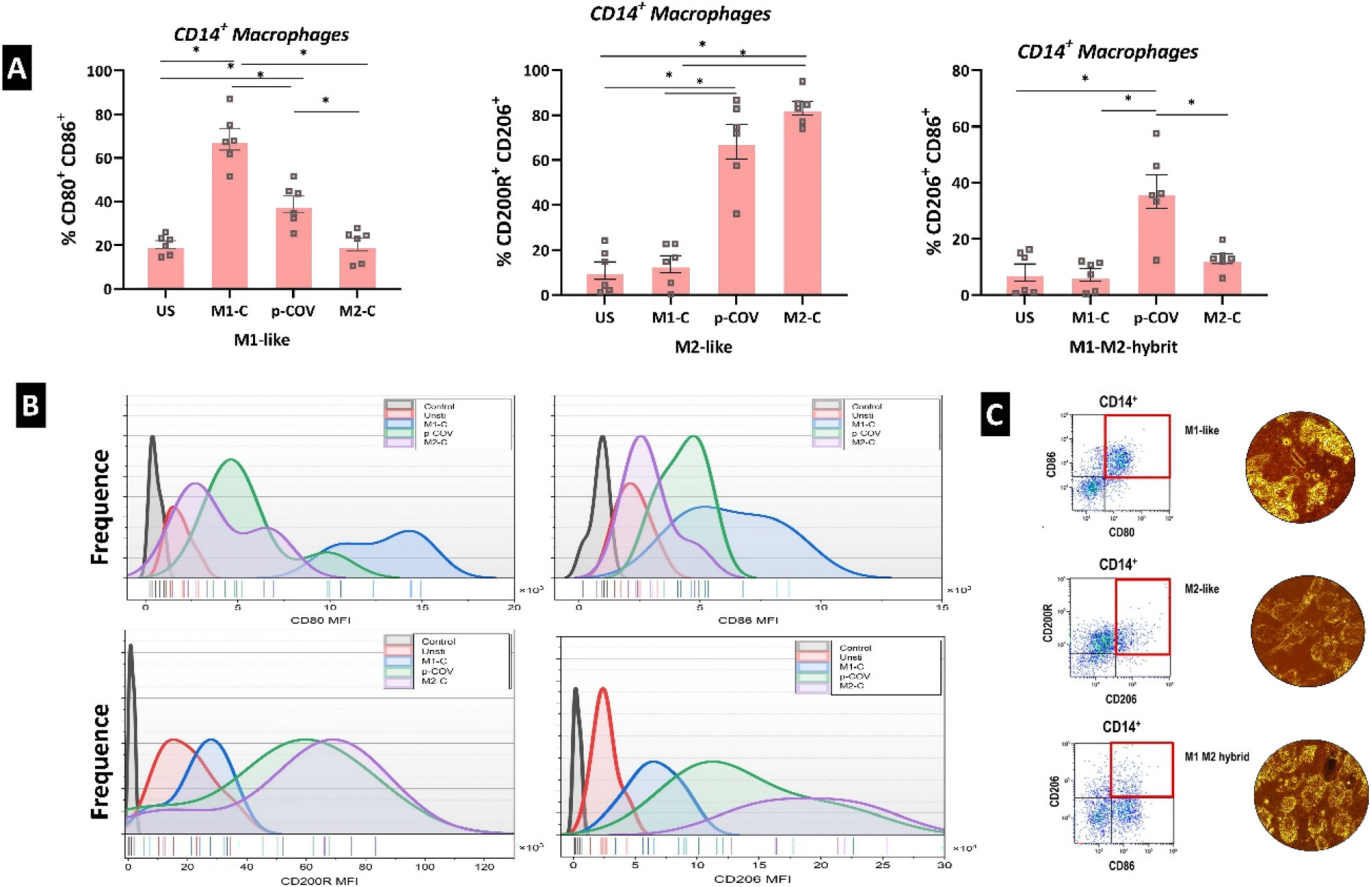
Flow cytometry analysis results unveiled the presence of hybrid macrophages in pCOV. A) Percentage values of macrophage subgroups post-FC analysis. B) Mean fluorescence intensity (MFI) values of M1 and M2 key markers in FC. C) Microscopic view through phase-contrast depicting M1, M2, and hybrid macrophages. ‘*’ denotes statistical significance at p<0.005. For multiple comparisons, ANOVA followed by post hoc Tukey’s HSD test was applied.

Co-culture studies revealed that the pCOV occupied an intermediate position between the M1 control and M2 control. Notably, the pCOV displayed a significantly higher proportion of the M1-M2 hybrid (CD206^+^CD86^+^) phenotype compared to other groups. According to the analyses, the M1/M2 (CD80^+^CD86^+^/CD200R^+^CD206^+^) ratio was higher in the unstimulated and M1 control groups, while the M2/M1 ratio was found to be more prevalent in the p-COV and M2 control groups (Figure 6 and Supplementary Figure 7).

### Similar cytokine and chemokine correlation between the pCOV and critical COVID-19 cases

**Figure 7:**
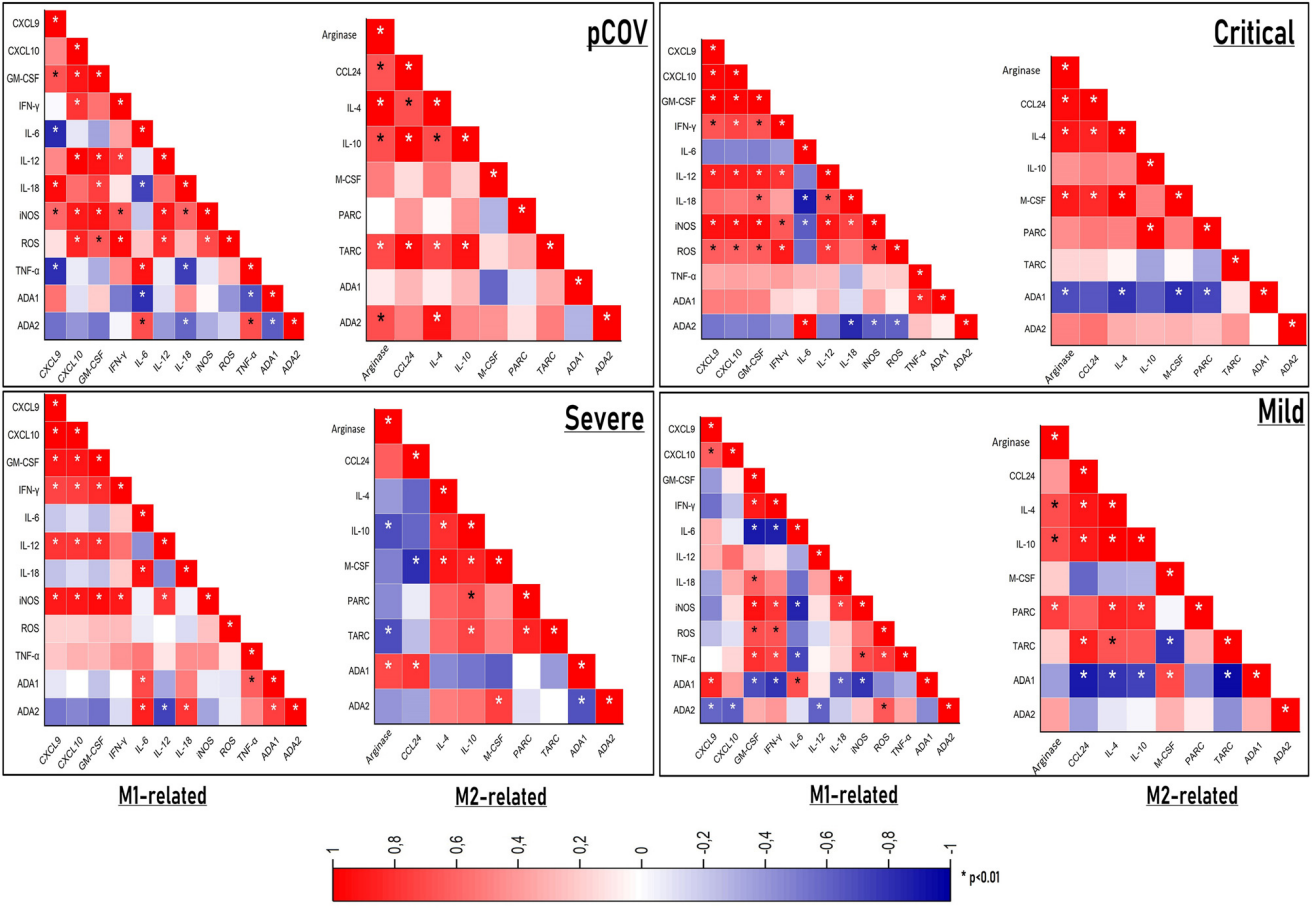
The correlation between ADA1 and ADA2 activity and M1 and M2 related factors indicated that p-COV exhibited a profile similar to critical and severe cases*.* According to the Pearson correlation test, significance (*) was set at p<0.01, and correlation coefficients (R) between 0 and 0.3 were categorized as a weak correlation, values between 0.3 and 0.7 as a moderate correlation, and values between 0.7 and 1 as a strong correlation.

In the correlation analysis, it was observed that the pCOV displayed a resemblance to critical and severe COVID-19 cases, and the profiles of cytokines and chemokines were consistent with each other. In pCOV and critical COVID-19 cases, ADA2 showed a positive correlation with Arginase, CCL24, IL-4, IL-10, M-CSF, PARC, and TARC. Likewise, a comparable profile was noted in severe cases, with the exception of Arginase and CCL24. Intriguingly, M2-related factors exhibited an inverse correlation with ADA1 in both critical and severe cases (excluding Arginase and CCL24) (Figure 7).

### Functional and clinical validation of the ADA2–M2 macrophage axis in COVID-19

**Figure 8:**
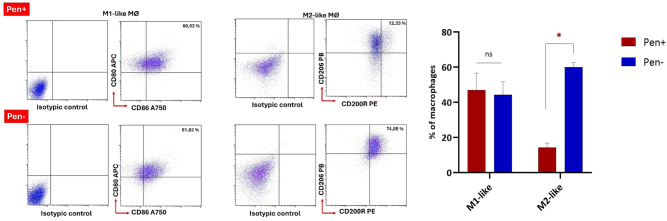
Under *in vitro* co-culture conditions, the addition of pentostatin (Pen) led to total ADA (tADA) inhibition, which did not affect M1-like macrophages but significantly impacted M2-like macrophages.

In COVID-19, particularly in severe and fatal cases, the observed positive correlation between ADA2 and M2-like macrophages has so far been based solely on protein expression levels and flow cytometry data. Therefore, to evaluate the effects of strong inhibition of ADA1 and ADA2 on macrophages, a chemotherapeutic agent called pentostatin was used. In this context, as previously described, co-culture models were established to assess the impact of pentostatin on macrophages. While M1-like macrophages appeared to be almost completely unaffected by pentostatin and ADA inhibition, a significant reduction in M2-like macrophages was observed (Figure 8). We similarly employed EHNA for tADA inhibition, but ultimately observed negligible effects on macrophage polarization and total cell populations.

To determine whether these mechanistic findings were reflected at the clinical level, we performed multivariable logistic regression analyses in the patient cohort. In multivariable logistic regression analysis adjusting for age, sex, CRP, ferritin, and D-dimer, ADA2 remained independently associated with mortality (OR=1.167, 95 % CI: 1.035–1.317, p=0.012), indicating that the observed relationship was not solely driven by demographic or inflammatory confounders. ADA2 alone demonstrated moderate discrimination in cross-validated analysis (AUC≈0.776 ± 0.08). Incorporation of clinical and inflammatory covariates improved model performance (AUC≈0.808 ± 0.09), suggesting additive predictive value beyond ADA2 alone. Multivariable logistic regression analysis and cross-validated ROC curves are shown in Supplementary Table 2 and Figure 8.

### Laboratory parameter profiles of COVID-19 groups

In our study, we noted a decrease in the levels of monocytes, lymphocytes, and platelets as the disease progressed, accompanied by an increase in ferritin, procalcitonin, CRP, and D-Dimer levels. Severe cases exhibited the highest values for AST and ALT liver enzymes, while a significant elevation was observed in LDH levels among critical patients (Supplementary Figure 9).

## Discussion

Alterations in the numbers of monocytes/macrophages during SARS-CoV-2 infection are closely linked to the disease’s severity [43]. However, which types and characteristics of macrophages exert an influence on the severity of the disease? While there have been speculations regarding the predominant macrophage types in COVID-19 cases, conclusive evidence is still lacking. Some studies suggest that macrophages of the M1 phenotype may impact the disease prognosis due to elevated levels of cytokines and chemokines associated with M1 [44], 45]. Conversely, other studies propose a connection between M2 macrophages and the frequently observed pulmonary fibrosis in cytokine storm scenarios [46]. However, there is a limited number of studies in the literature focusing on the subtypes of macrophages. A particular study demonstrated dense infiltration of M1 macrophages, monocytes, and neutrophils in lung biopsies [47]. Conversely, another study, highlighting the plasticity of macrophages in COVID-19, provided evidence suggesting that the pronounced infiltration of macrophages may play a role in directing them toward the M2 phenotype [48]. In a separate study, expression analyses of peripheral blood-derived monocytes were conducted in COVID-19 patients [49]. It was revealed that these monocytes could express cytokines characteristic of both M1 and M2 macrophages. To the best of our knowledge, this research represents the initial investigation into ADA-mediated macrophage polarization in the context of SARS-CoV-2 infections. In this study, we found integrated clinical and mechanistic evidence suggesting that ADA isoenzyme activity is associated with COVID-19 severity and appears to parallel shifts in macrophage polarization within the disease microenvironment. ADA2 showed a stronger association with advanced disease stages, while *in vitro* analyses supported a potential involvement of ADA-related pathways in macrophage polarization dynamics under COVID-19-related conditions.The integration of severity-stratified clinical data with mechanistic experiments conducted across two independent clinical centers provided a coherent and well-characterized framework that reinforced the robustness of the findings and underscored their potential translational relevance.

Numerous studies have investigated the role of ADA in various inflammatory diseases. In one study, higher tADA and ADA2 activities were observed in patients with autoimmune diseases such as systemic lupus erythematosus (SLE), myasthenia gravis (MG), ankylosing spondylitis (AS), and rheumatoid arthritis (RA) when compared to healthy controls. This increase in ADA enzyme activity has also been suggested as a potential marker for MAS [50]. Furthermore, in tuberculosis pleural effusion (TPE) patients, an elevation in pleural ADA and isoenzyme activities, with ADA2 constituting the majority of tADA activity, underscores its diagnostic significance [51]. In a separate study examining plasma ADA1 and ADA2 activity in various cancer types, higher ADA2 activity was identified in cancer patients. The study suggested that ADA1 and ADA2 may exhibit distinct roles, with opposing functions, and proposed a potential protective role for ADA2 in cancer [52]. In another study analyzing ADA activity in relation to the severity of COVID-19, it was noted that ADA activity was significantly elevated in severe cases compared to mild COVID-19 cases and the healthy control group [53]. Our study similarly observed increased tADA activity, as well as ADA1 and ADA2 isoenzyme activities, corresponding to the severity of the disease. Given the abundant presence of ADA in lymphoid tissues, it can be inferred that enzyme activation intensifies in conditions characterized by heightened inflammation.

MAS, characterized by a hyperinflammatory state cytokine storm, represents a life-threatening condition with the potential to induce multiple organ failure. The association between ADA and MAS is frequently observed, particularly in rheumatological diseases. In an investigation focusing on immune-mediated disorders, comparative analyses were conducted on ADA2 activity and MAS findings. The study revealed elevated ADA2 activity in patients with systemic juvenile idiopathic arthritis (JIA) compared to healthy controls. Further analyses based on MAS markers indicated higher ADA2 levels in JIA patients with MAS compared to those without MAS findings, suggesting ADA2 as a reliable marker for MAS with a high confidence interval. Moreover, when examining other MAS markers, a correlation was identified between IL-18, IFN-γ, CXCL9, ferritin, and ADA2 activity [54]. In another study exploring serum ADA levels in patients with hemophagocytic lymphohistiocytosis (HLH), lymphoma-associated hemophagocytic syndrome (LAHS), infection-associated hemophagocytic syndrome (IAHS), and MAS, significantly elevated ADA levels were observed across all patient groups compared to healthy controls [55]. Cytokine storms and MAS-like hyperinflammation are frequently observed in the deterioration of COVID-19 conditions [56]. This study identified a positive correlation between ADA2 activation and the progression of the disease. Particularly in critical cases, ADA2 activation exhibited a more than threefold increase compared to the healthy control group. Moreover, a significant positive correlation between ADA2 levels and mortality was consistently observed in both severe and critical cases (Supplementary Figure 10). Taking into account the reported connections between ADA2 and macrophage activation syndrome (MAS), along with the observed elevation of MAS markers in severe/critical COVID-19 cases, underscores the crucial role of ADA2 in the cytokine storm associated with COVID-19 and its consequential fatalities.

The M1/M2 polarization paradigm has been a significant focus of research in recent years. In the mixed or hybrid phenotype, macrophages express characteristics of both the monocyte/macrophage subgroup, exhibiting not only pro-inflammatory properties but also contributing to tissue repair [57]. Furthermore, as previously observed in the lung and peritoneal cavity, hybrid macrophages can form with tissue-specific variations [58]. Hybrid macrophage formation is observable not only at the population level but also at the single-cell level [59]. It has also been shown to exist in certain pathological conditions, such as some cancers and autoimmune diseases [60]. In a study conducted by Matic and colleagues, the assumption that non-canonical monocytes of M1 and M2 subtypes could mirror M1 and M2 macrophages led to the suggestion that SARS-CoV-2 infection might induce macrophages in the M1-M2 hybrid phenotype [57]. Considering that pulmonary fibrosis and cytokine storm are deemed significant risk factors for mortality in severe cases of COVID-19, the recruitment of hybrid macrophages with pro-inflammatory fibrosis potential seems plausible. The results obtained from the pCOV established in this study, characterized by a severe and critical case-like profile, reveal the formation of macrophages in a hybrid phenotype.

The potential role of ADA in macrophage polarization in the context of COVID-19 remains unexplored. Intriguingly, correlation analyses revealed associations between ADA isoenzymes and M1-and M2-related cytokines, chemokines, and other factors. Correlation analyses revealed that the pCOV bore similarities to severe and critical cases, though not identical. Firstly, in the examination of M1-related factors, it was observed that the majority of these factors were inversely correlated with ADA2 in both severe and critical cases and the pCOV. It is not unexpected that IL-6 and TNF-α, crucial players in the inflammation process in COVID-19, emerged as two exceptions in all three groups. In mild and presymptomatic cases, no evidence of a relationship between ADA2 and M1-related factors was found. Secondly, when exploring the relationship between M2-related factors and ADA2, the findings from critical cases and the pCOV were largely consistent, indicating a positive correlation between ADA2 and M2-related factors. Moreover, in severe cases, ADA2 exhibited positive correlations with IL-4, IL-10, and M-CSF. The major results derived from the correlation analyses are as follows: i) the observation that ADA2 and M2-related factors are positively correlated, particularly with the severity of the disease; ii) the discovery that the generated pCOV is akin to severe and critical cases; iii) no association between ADA2 and M2 was observed in mild and presymptomatic cases. These results suggest an implication that ADA2 is associated with M2 macrophages in SARS-CoV-2 infections. On the other hand, FC analyses revealed the presence of macrophages with an M1-M2 hybrid phenotype in the pCOV. This finding, combined with the increase in both M1 and M2-related factors in critical and severe cases, appears consistent. However, ADA2 displayed an inverse correlation with M1-related factors in the critical, severe, and pCOV. When evaluated collectively, hybrid macrophages can be considered as M2-like macrophages that may exhibit pro-inflammatory properties, reflecting the inflammatory environment. It does not appear unreasonable to posit that with the escalation in disease severity, there is an increase in ADA2 activity, leading to a shift in macrophage tendencies from M1 to a hybrid state, eventually transitioning towards M2.

Pentostatin is a purine analog and a chemotherapeutic agent that inhibits the enzyme adenosine deaminase (ADA1 and ADA2). Our findings indicate a strong positive correlation between M2-like macrophages and ADA2 enzyme activity. To investigate the biological basis of this correlation and to clarify the relationship between ADA2 and M2 macrophages in the context of COVID-19, we aimed to assess the potential effects of inhibiting ADA enzyme activity (both ADA1 and ADA2) on macrophages. For this purpose, we employed pentostatin, which is known for its dual inhibitory effect on both ADA1 and ADA2. Interestingly, while M1-like macrophages were not significantly affected by pentostatin, the proportion of M2-like macrophages was markedly reduced (by 12.33 %) in its presence compared to the control. This observation provides important evidence supporting a direct association between ADA2 activity and M2-like macrophages, particularly in severe cases of COVID-19.

An important aspect of our findings is that ADA2 retained independent prognostic relevance in multivariable regression analysis. Even after adjustment for age, sex, and key inflammatory markers, ADA2 remained significantly associated with mortality, and ADA2 alone demonstrated moderate discriminatory capacity. These results suggest that ADA2 is not merely a surrogate of systemic inflammation but reflects a distinct immunometabolic signal within the COVID-19 microenvironment. At the same time, ADA2 activity likely operates within a broader biological context shaped by host-specific factors. Unmeasured influences – such as concomitant medications, genetic variability in purinergic signaling pathways, or comorbid conditions not fully captured in our dataset – may further modulate its activity and clinical impact. Future studies integrating comprehensive clinical phenotyping with mechanistic investigation will be essential to clarify the precise pathways through which ADA2 contributes to COVID-19 severity.

Human macrophages can exhibit varying expression profiles under different conditions and stimuli. Interestingly, contrary to findings in mice, results indicate that arginase and iNOS production in human macrophages is limited. Some authors demonstrate that these markers cannot be produced by monocyte/macrophage populations in response to various stimuli, while others suggest that macrophages can express arginase or iNOS, particularly in scenarios like trauma, stress, and inflammation [61]. Additionally, human T cells have shown a limited capacity to produce iNOS and arginase [62]. In this study, both markers were observed in co-culture environments, with notably higher iNOS production in the p-COV and M1-control groups compared to the M2 group. Although it remains unclear whether this increase is associated with T cells, macrophages, or auto-serums added to the co-culture environment, the variations in both markers are particularly intriguing.

Overall, our study reveals an increase in ADA1 and ADA2 enzyme activity in correlation with the severity of COVID-19. Analysis of M1-and M2-related cytokines, chemokines, and other molecules demonstrated a significant elevation in severe and critical cases. Furthermore, in severe and critical cases, there was a positive correlation observed between ADA2 and M1-and M2-related cytokines and chemokines. This correlation was also evident in the pCOV. Notably, severe and critical COVID-19 cases, along with the pCOV, exhibited a remarkably similar M1/M2 signature. FC evaluations indicated significantly lower levels of M1-like macrophages (CD80^+^CD86^+^) in the pCOV compared to the M1 control, while M2-like macrophages (CD206^+^CD200R^+^) showed no statistically significant difference from the M2-control group. Interestingly, our *in vitro* analysis revealed a significantly higher presence of M1-M2 hybrid macrophages (CD86^+^CD206^+^) in the COVID-19 group compared to both the M1- and M2-control. According to all our analyses, it was found that despite a trend towards M2 polarization in macrophages in severe cases of COVID-19, the obtained macrophages exhibited a hybrid population with characteristics from both M1 and M2 phenotypes. The relationship between ADA2 and M2-like macrophages has also been validated through experiments involving the use of pentostatin. Comparing healthy, a/presymptomatic, and mild COVID-19 cases, it becomes apparent that severe COVID-19 cases are associated with a favorable prognosis. We speculate that the improvement of prognosis in severe COVID-19 cases is associated with the decrease of ADA2 activity and the shift of M1-M2 macrophage hybrid phenotype to M1, while the worsening of prognosis is associated with the increase of ADA2 activity and the shift to M2 phenotype, considering healthy, a/presymptomatic, and mild COVID-19 cases.

## Strength and limitations

A key strength of this study lies in its integrated approach, combining severity-stratified clinical data with controlled *in vitro* experiments to evaluate the relationship between ADA2 activity and macrophage polarization in the context of COVID-19. This dual perspective allowed for a structured assessment of ADA isoenzyme dynamics in relation to immune polarization. By specifically focusing on ADA2-mediated macrophage modulation within a defined SARS-CoV-2–related inflammatory setting, the study offers a clinically relevant contribution to the existing literature. However, we acknowledge certain limitations in our study. Firstly, an increased number of cases, particularly those representing critical conditions, could have enhanced the robustness of our findings. Additionally, the exclusion of functional parameters in the analysis of macrophage subgroups represents a noteworthy limitation. The incorporation of markers such as ROS and NO, or the inclusion of a phagocytosis test step in FC analyses, could have provided insights into the functions of identified macrophages, thereby strengthening the data. In addition, CD69 expression used to define lymphocyte activation reflects early activation and may include bystander responses within the inflammatory serum-conditioned co-culture environment rather than exclusively antigen-specific T-cell responses. Furthermore, integrating the distribution of non-classical M1 and M2 monocyte subsets in relation to ADA2 activity could have provided additional mechanistic depth and strengthened the interpretation of the macrophage–ADA2 axis. On the other hand, multivariable analyses were performed to adjust for age and other clinically relevant covariates, we acknowledge that residual age-related confounding cannot be entirely excluded due to the cross-sectional study design and the absence of fully age-matched controls. Lastly, measuring the presence of adenosine/deoxyadenosine in patients could offer detailed information about substrate accumulation in different case groups, thereby making a valuable contribution to the study.

## Conclusions

The findings obtained in this study regarding the relationship between ADA2 and macrophage polarization may gain further significance as the mechanistic pathways underlying inflammatory responses become more precisely defined. Within this context, the observed associations may contribute to a more refined understanding of immune dysregulation in severe infections. These insights have potential implications for improving risk stratification and immune monitoring strategies in clinical practice. A deeper understanding of ADA-related immune modulation may facilitate the development of more precise, mechanism-driven therapeutic strategies and contribute to improved clinical management of severe inflammatory conditions. Future research should prioritize longitudinal evaluation of ADA isoenzyme dynamics and further clarify their mechanistic and potential therapeutic roles in inflammatory conditions.

## Supplementary Material

Supplementary Material
