# Supplementary material for: The dynamic interplay of M1/M2-like macrophages and adenosine deaminase activity modulates COVID-19 severity
Source: Open Med (Wars). 2026 May 4;21(1):20261429. doi: 10.1515/med-2026-1429 (PMC13135473; doi:10.1515/med-2026-1429)
Supplement: Supplementary file 1 — Supplementary Material [file j_med-2026-1429_suppl_001.docx]

**Supplementary data legends**

***Supp. Tab.1.* Examination of demographic data in relation to the course of COVID-19.** ^a1^ A/pre-symptomatic vs Mild, Severe and Critical, ^a2^Healthy Control vs Mild, Severe, Critical,^b1^ A/pre-symptomatic vs Severe, ^b2^ A/pre-symptomatic vs Mild, ^c1^Severe vs Mild, ^c2^Severe vs A/pre-symptomatic, ^c3^ Critical vs A/pre-symptomatic, ^c4^ Severe vs Critical, ^c5^ Mild vs Critical

***Supp. Tab.2.* Multivariable logistic regression model including ADA2, D-dimer, sex, age, ferritin, and CRP.** Odds ratios (OR) are reported with 95% confidence intervals (CI) and p-values.

***Supp.Fig.1A* Optimizing EHNA for the measurement of ADA1 and ADA2 enzyme activity**

***Supp.Fig.1B* Optimizing pentostatin for the measurement of ADA1 and ADA2 enzyme activity**

***Supp.Fig.2* Monocyte isolation protocol utilizing Percoll density gradient centrifugation**

***Supp.Fig.3* Analysis of SARS-CoV-2-specific lymphocyte activation following stimulation with p-COV.**

***Supp.Fig.4* Assessment of monocyte purity isolated using Percoll.**

***Supp.Fig.5* Proportions of ADA1 and ADA2 in the total ADA activity across COVID-19 patient groups.**

***Supp.Fig.6* Proportions of ADA1 and ADA2 in the total ADA activity in pCOV.**

***Supp.Fig.7* The distribution of macrophages in pCOV reveals a proportional pattern.**

**Supp. Fig. 8. ROC analysis with 5-fold cross-validation for mortality prediction*.*** *ROC curves showing the predictive performance of ADA2 alone (red) and the multivariable model including ADA2 plus clinical covariates (blue) for mortality. The diagonal dotted line represents random classification (AUC = 0.5). Optimal thresholds were determined using the Youden index.*

***Supp.Fig.9* Analyzes of laboratory findings in COVID-19 patient groups**. ‘*’ denotes statistical significance at p < 0.005.

***Supp.Fig.10.* ADA2-associated mortality rates in severe and critical COVID-19 cases.** Probability of death rate is expressed as a percentage. The statistical significance level is p<0.005.

|  | A/pre-symptomatic (n=37) | | Mild  (n=36) | | Severe  (n=41) | | Critical  (n=6) | | Total COV-19  (n=120) | | HC  (n=40) | | p value | |
| --- | --- | --- | --- | --- | --- | --- | --- | --- | --- | --- | --- | --- | --- | --- |
| Sex assigned at birth (%) | M | F | M | F | M | F | M | F | M | F | M | F | 0,938 |  |
|  | 20  (54) | 17  (45,9) | 19  (52,7) | 17  (47,3) | 22  (53,7) | 19  (46,3) | 4  (66,7) | 2  (33,3) | 65  (54,2) | 55  (45,8) | 16  (40) | 24  (60) |  |  |
| Age (mean±SD) | 45,40±16,56 | | 58,17±9,54 | | 59,01±14,22 | | 62,16±10,43 | | 54,68±14,93 | | 36,47±10,65 | | <0,01^a1, a2^ | |
| Comorbidty | | | | | | | | | | | | | | |
| Hypertension (%) | 1 (2,7) | | 5 (13,9) | | 11 (26,8) | | 1 (16,7) | | 18 (15) | | - | | 0,03^b1^ | |
| Diabetes mellitus (%) | 3 (8,1) | | 4 (11,1) | | 5 (12,2) | | 1 (16,7) | | 13 (10,8 ) | | - | | 0,902 | |
| Cardiovascular disease (%) | 3 (8,1) | | 3 (8,3) | | 4 (9,8) | | 1 (16,7) | | 11 (9,2) | | - | | 0,918 | |
| COPD (%) | 2 (5,4) | | 2 (5,6) | | 5 (12,2) | | 1 (16,7) | | 10 (8,3) | |  | | 0,568 | |
| Other (%) | 3 (8,1) | | 5 (13,9) | | 12 (29,2) | | 2 (33,3) | | 5 (18,3) | | - | | 0,221 | |
| Symptoms (first admission) | | | | | | | | | | | | | | |
| Fever (%) | 5 (13,5) | | 9 (25,0) | | 25 (61,0) | | 4 (66,7) | | 43 (35,8) | | - | | <0,01^c1,c2,c3^ | |
| Cough (%) | 4 (10,8) | | 20 (55,6) | | 23 (56,1) | | 5 (83,3) | | 52 (43,3) | | - | | <0,01^a1^ | |
| ARDS (%) | 4 (10,8) | | 15 (41,7) | | 27 (65,9) | | 5 (83,3) | | 21 (42,5) | | - | | <0,01^a1^ | |
| Myalgia (%) | 0 (0,0) | | 11 (30,6) | | 8 (19,5) | | 1 (16,7) | | 20 (16,7) | | - | | 0,005^b2^ | |
| Mechanical Ventilation (%) | - | | - | | 24  (58,5) | | 6  (100) | | 30  (25) | | - | | - | |

Supp. Tab.1

Supp. Tab.2

| **Variable** | **Odds Ratio** | **95% CI** | **p-value** |
| --- | --- | --- | --- |
| ADA2 | 1.090 | 1.022–1.162 | 0.009 |
| D-dimer | 1.014 | 0.937–1.097 | 0.337 |
| Sex (Male) | 0.688 | 0.200–2.363 | 0.552 |
| Age | 1.032 | 0.986–1.081 | 0.177 |
| Ferritin | 1.039 | 0.976–1.107 | 0.232 |
| CRP | 1.072 | 0.995–1.155 | 0.068 |


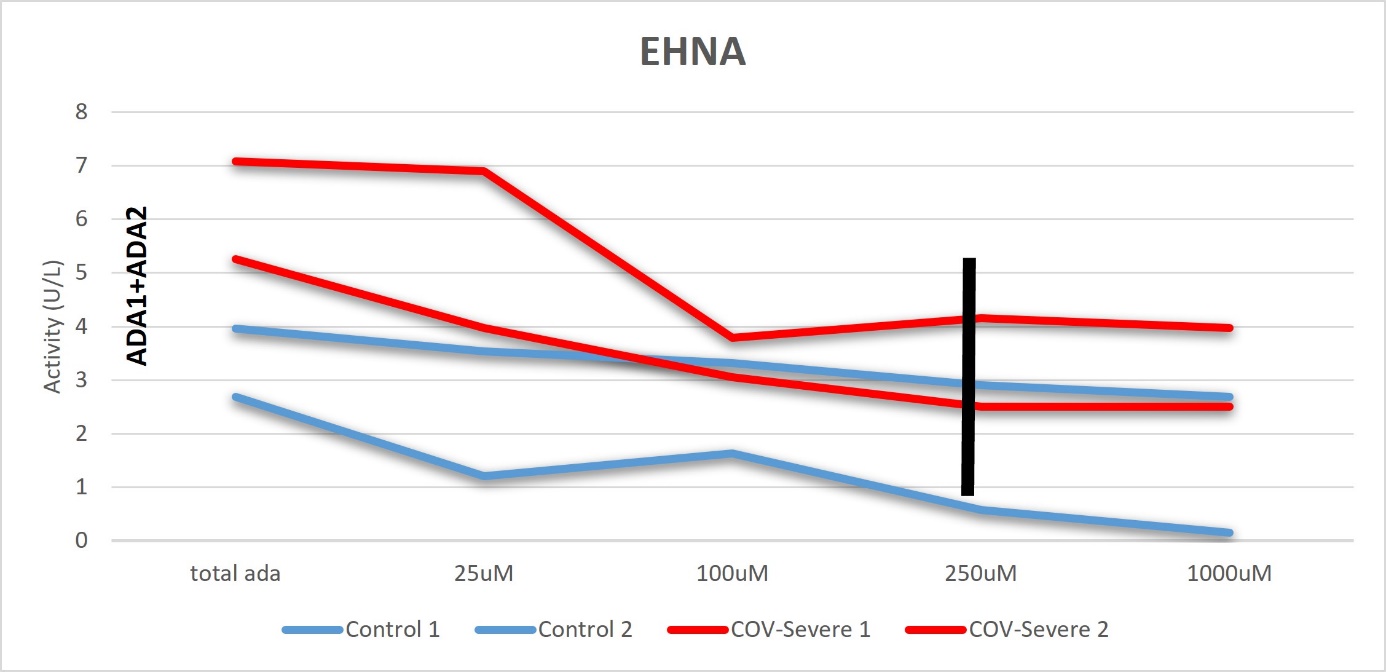


**A**


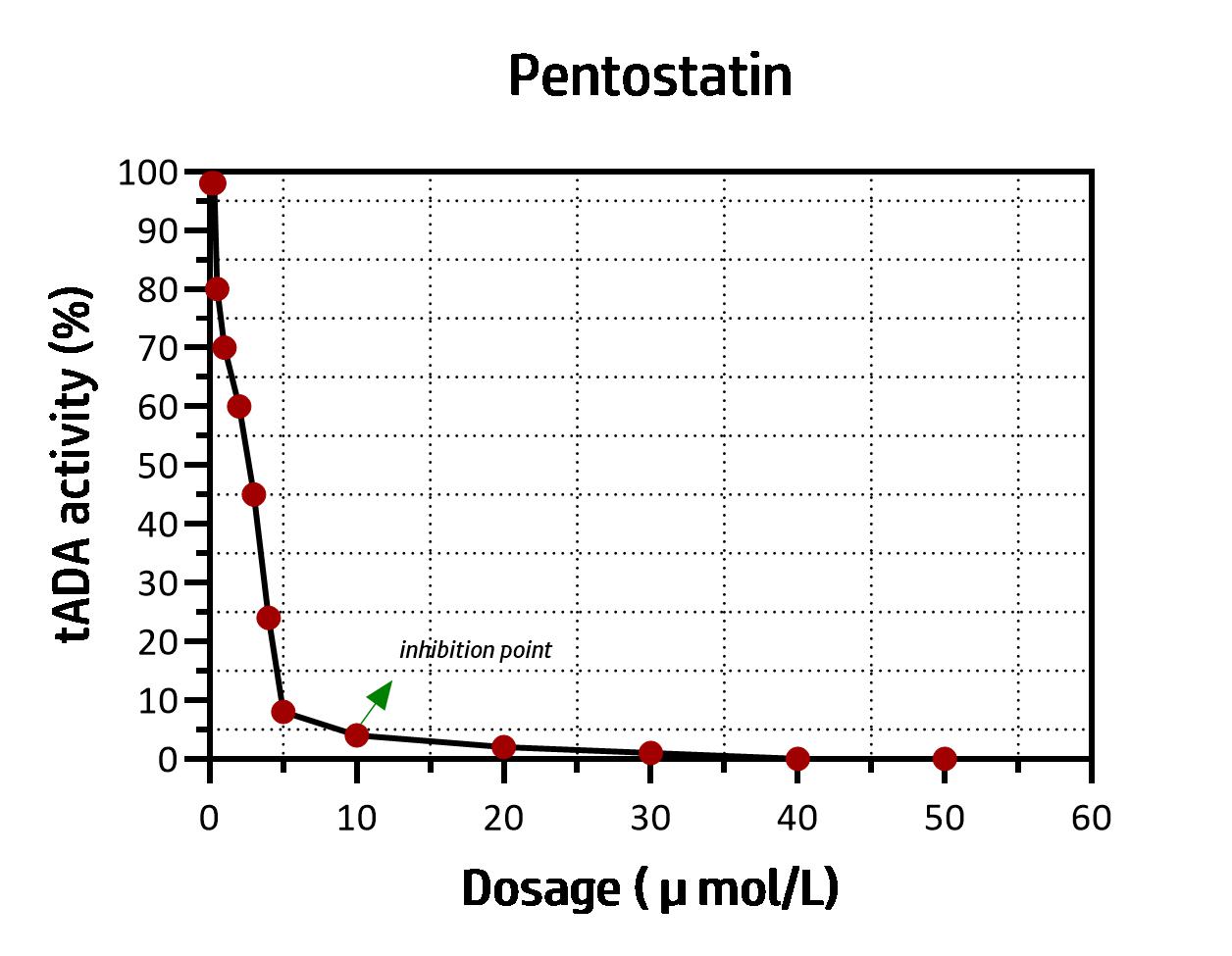


**B**

Supp. Fig.1

**
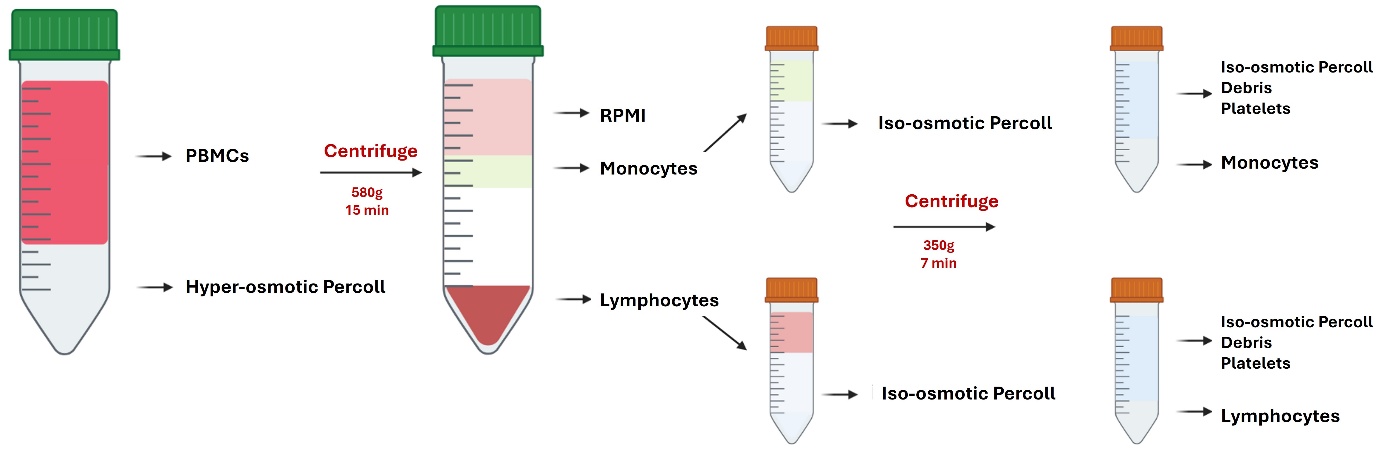
**

Supp. Fig.2


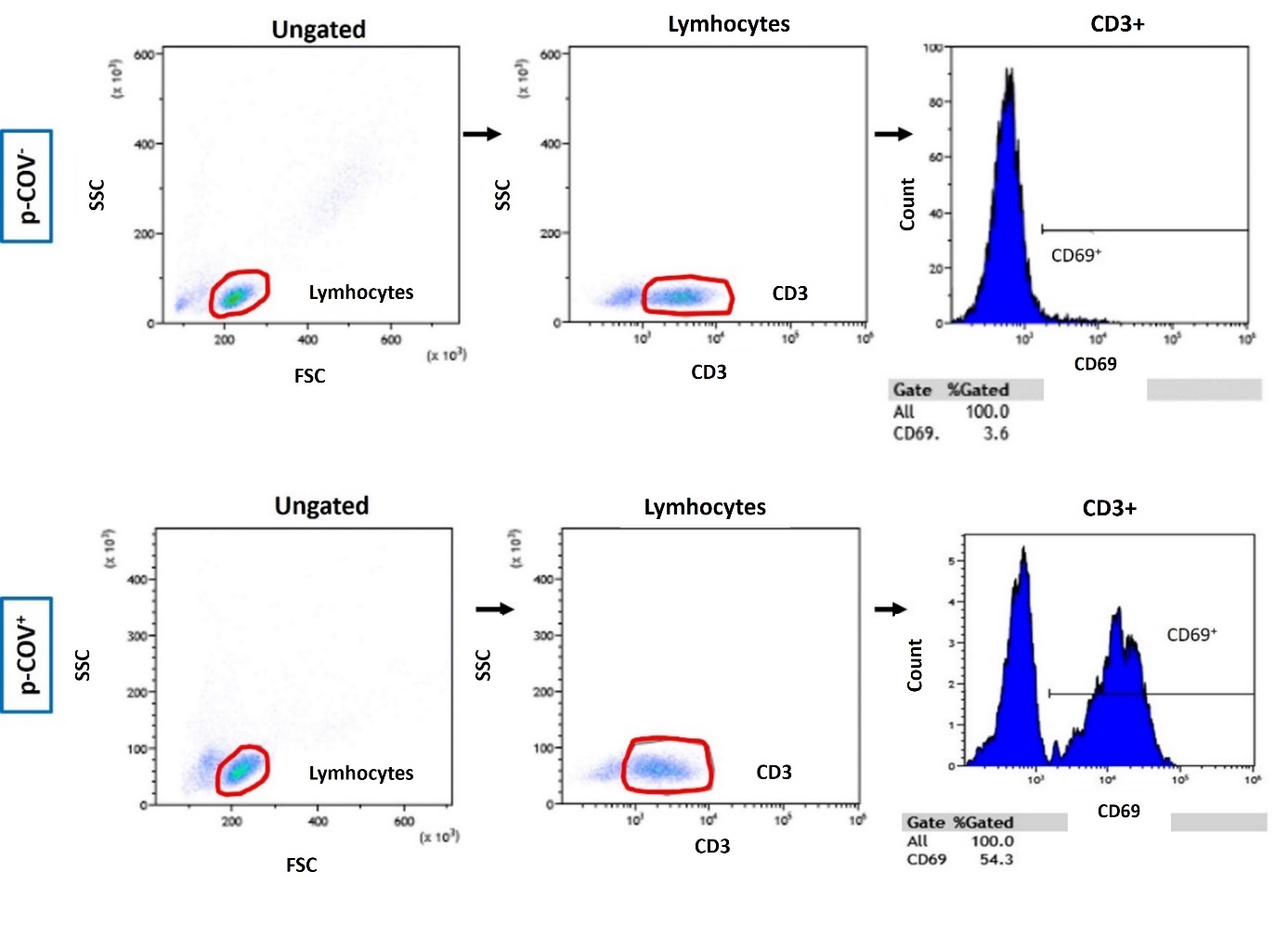


Supp. Fig.3


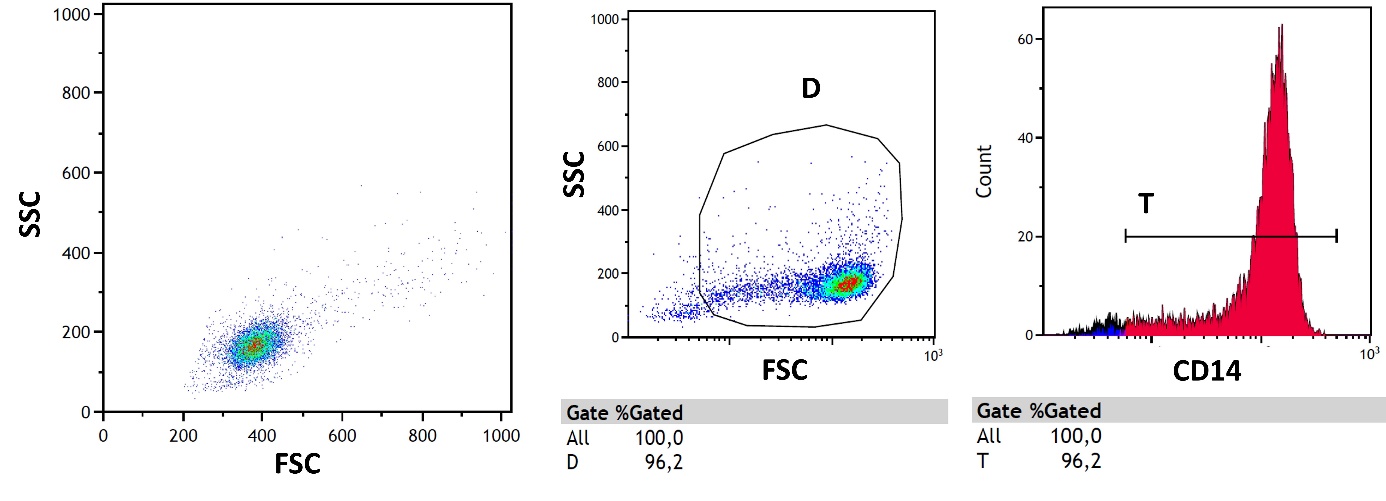


Supp. Fig.4


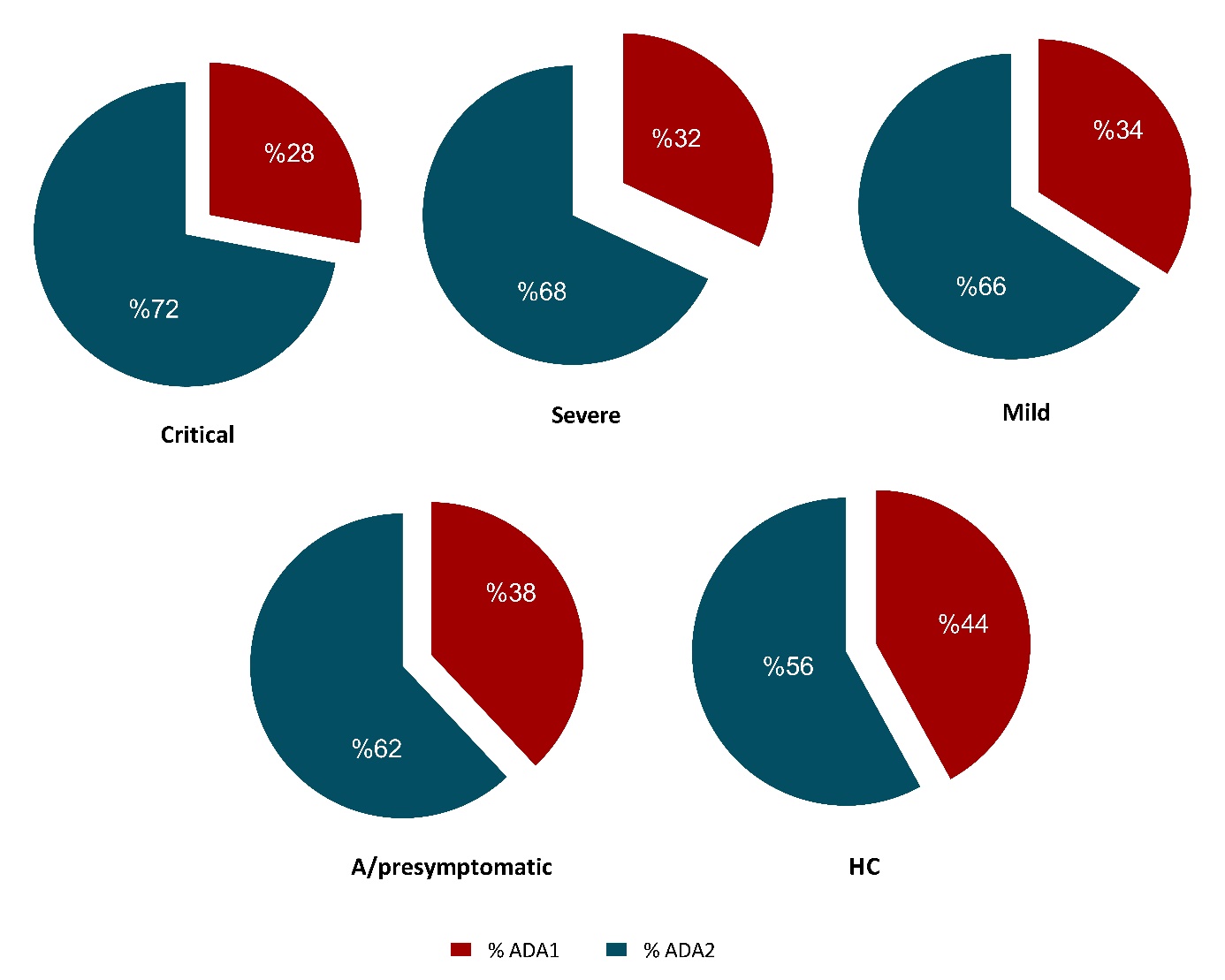


Supp. Fig.5


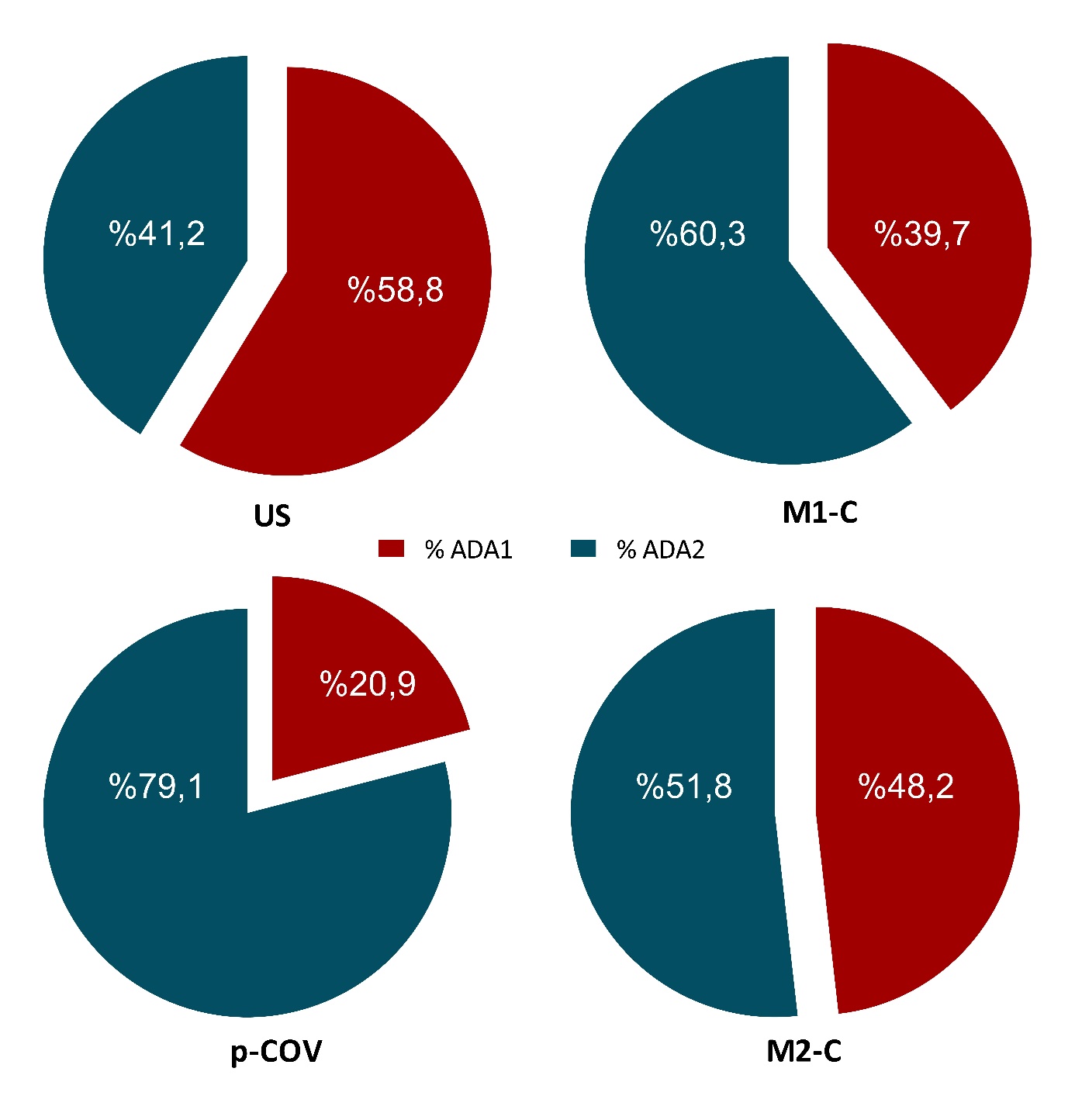


Supp. Fig.6


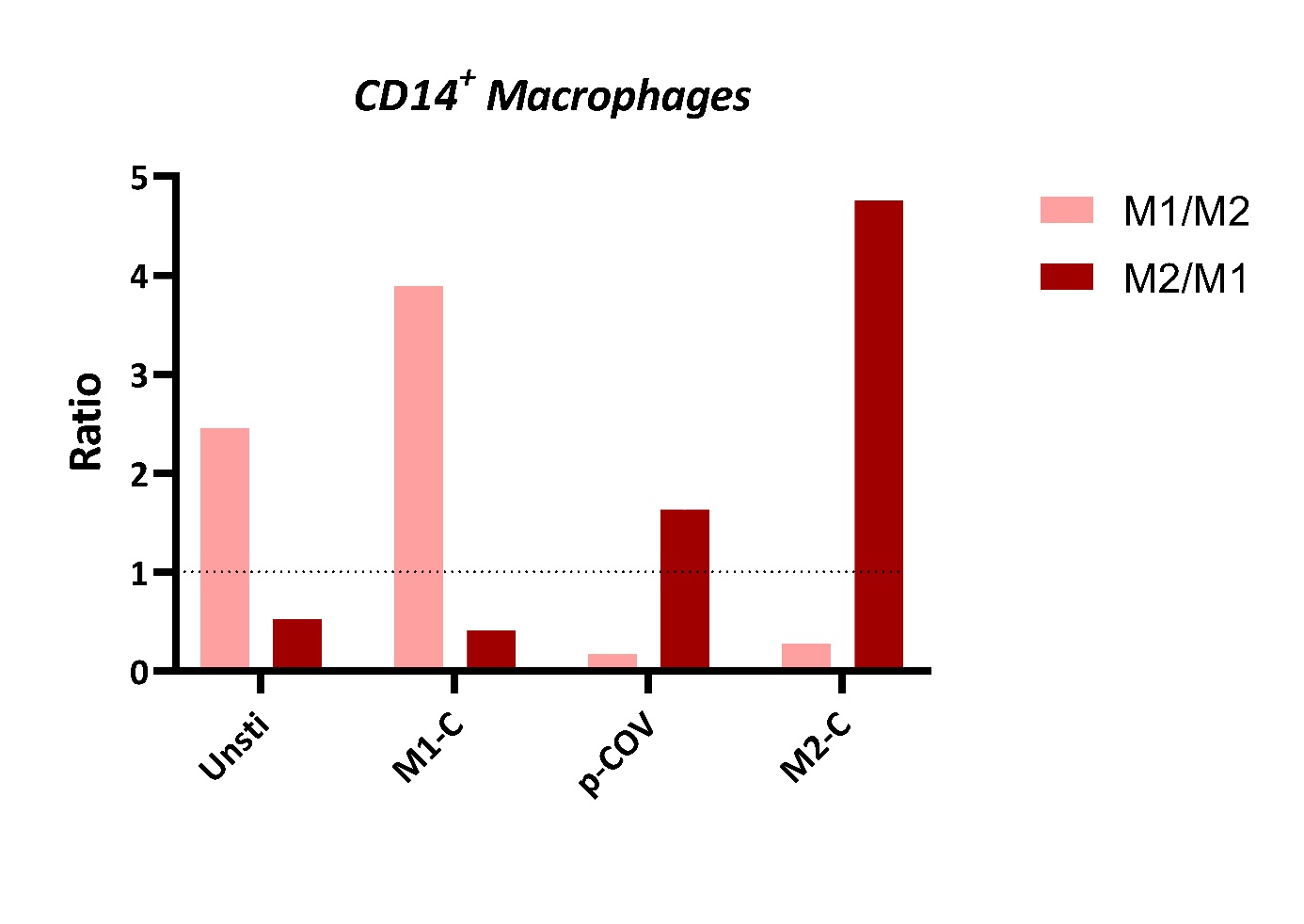


Supp. Fig.7


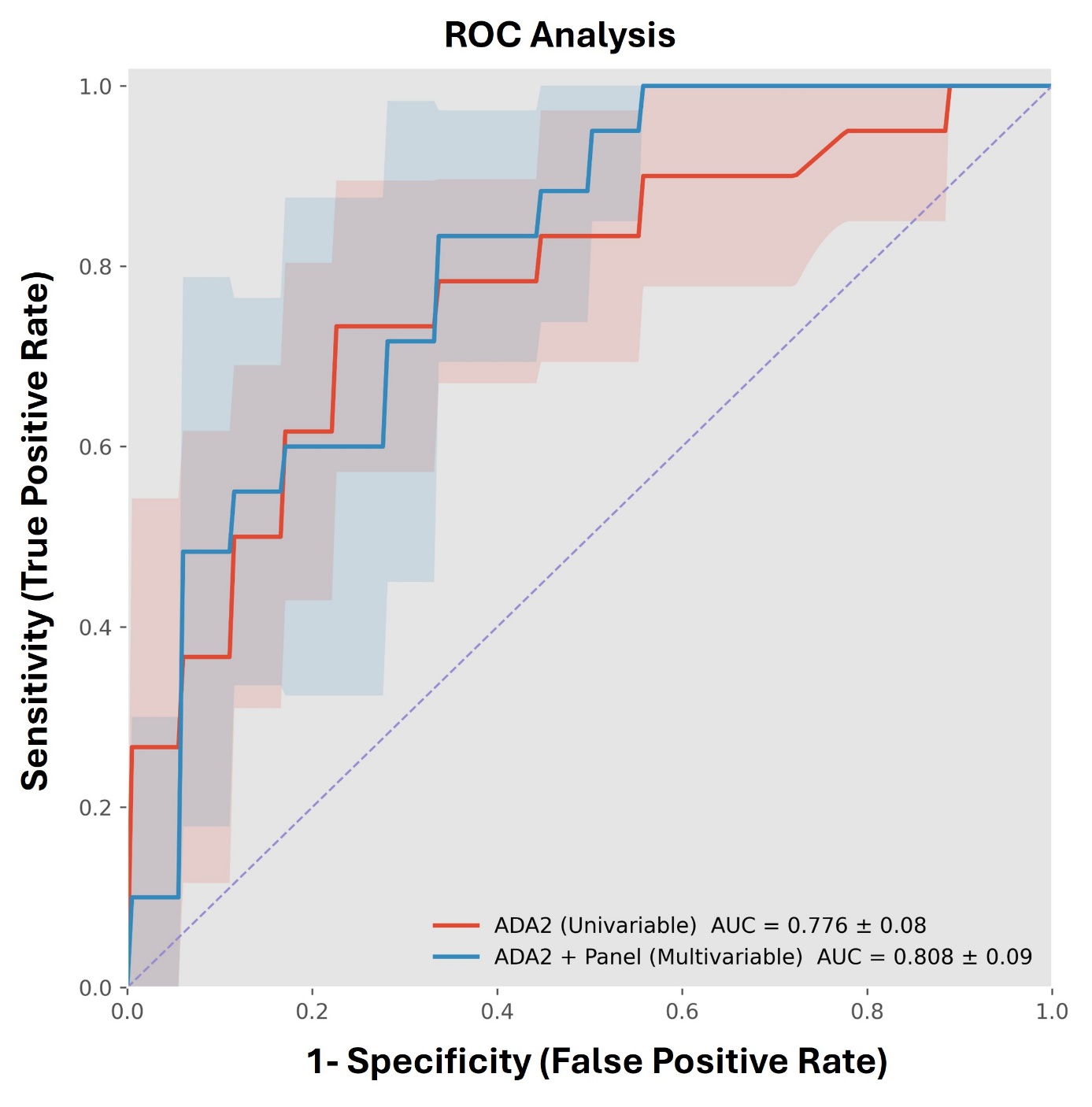


Supp. Fig.8


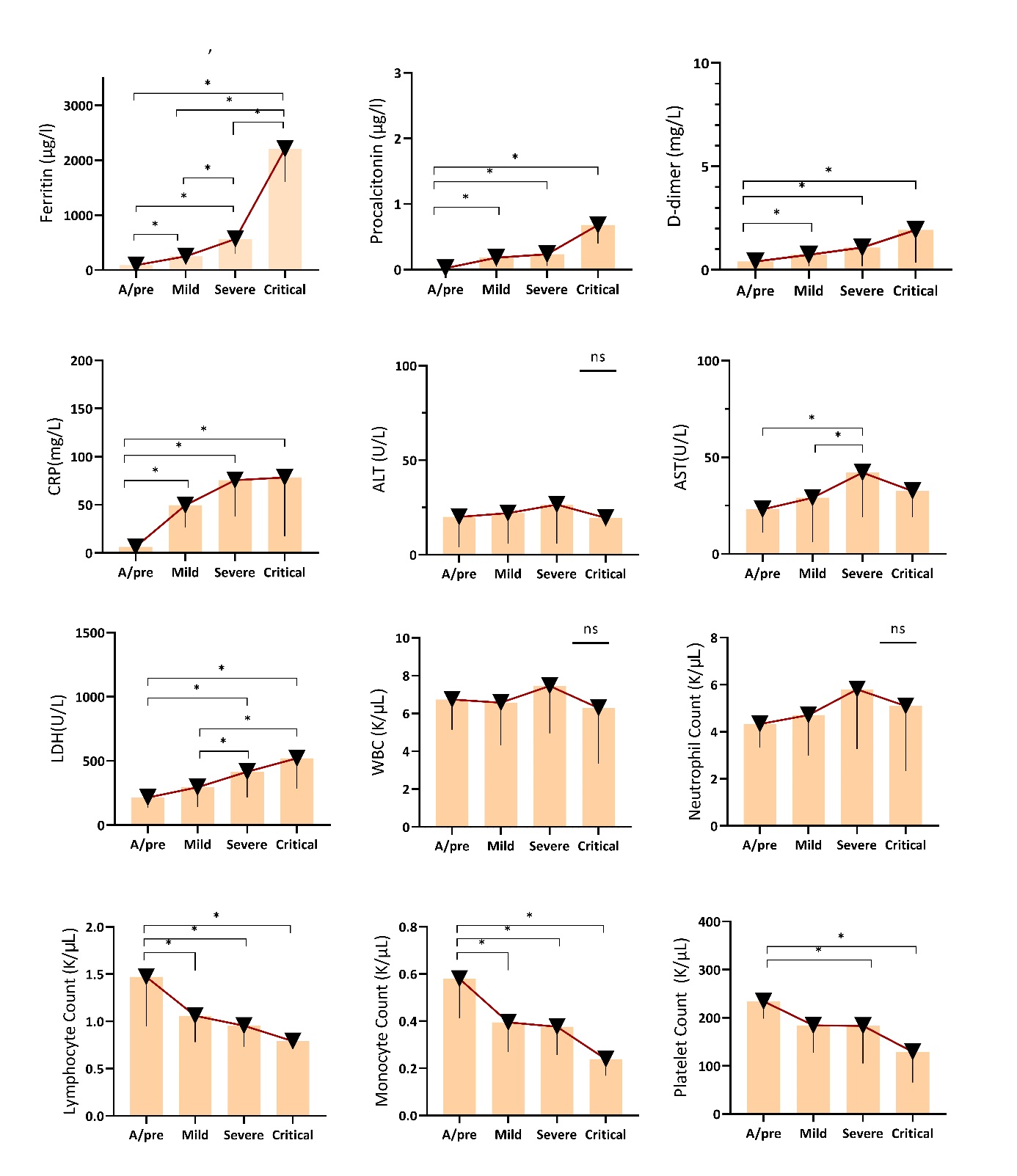


Supp. Fig.9


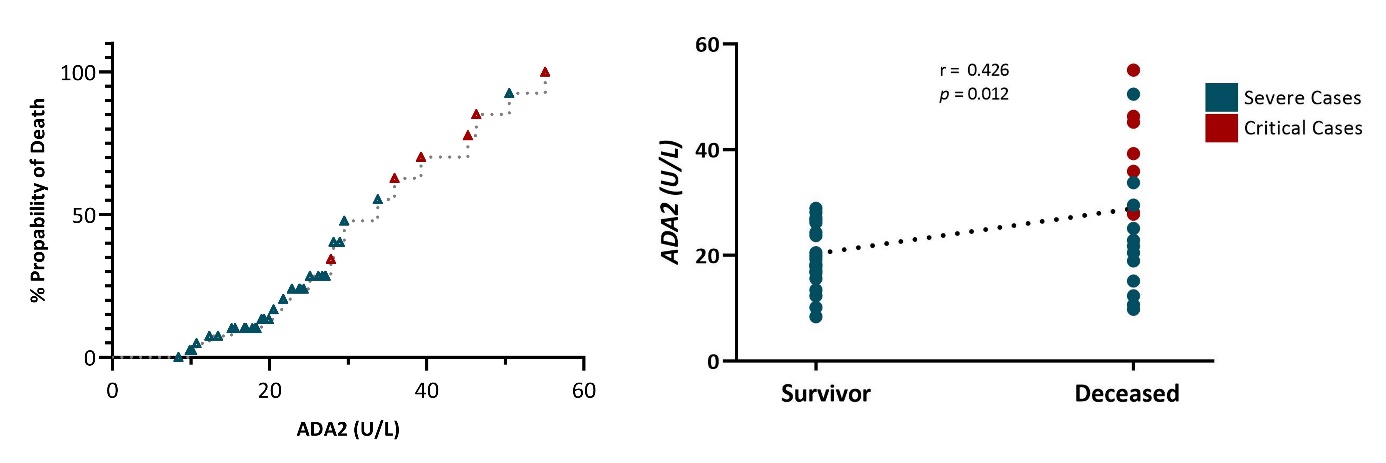


Supp. Fig.10
